# The association of lifetime alcohol use with mortality and cancer risk in older adults: A cohort study

**DOI:** 10.1371/journal.pmed.1002585

**Published:** 2018-06-19

**Authors:** Andrew T. Kunzmann, Helen G. Coleman, Wen-Yi Huang, Sonja I. Berndt

**Affiliations:** 1 Centre for Public Health, Queen’s University Belfast, Belfast, Northern Ireland, United Kingdom; 2 Division of Cancer Epidemiology and Genetics, National Cancer Institute, National Institutes of Health, Department of Health and Human Services, Bethesda, Maryland, United States of America; University of Toronto, CANADA

## Abstract

**Background:**

While current research is largely consistent as to the harms of heavy drinking in terms of both cancer incidence and mortality, there are disparate messages regarding the safety of light-moderate alcohol consumption, which may confuse public health messages. We aimed to evaluate the association between average lifetime alcohol intakes and risk of both cancer incidence and mortality.

**Methods and findings:**

We report a population-based cohort study using data from 99,654 adults (68.7% female), aged 55–74 years, participating in the U.S. Prostate, Lung, Colorectal, and Ovarian (PLCO) Cancer Screening Trial. Cox proportional hazards models assessed the risk of overall and cause-specific mortality, cancer incidence (excluding nonmelanoma skin cancer), and combined risk of cancer and death across categories of self-reported average lifetime alcohol intakes, with adjustment for potential confounders. During 836,740 person-years of follow-up (median 8.9 years), 9,599 deaths and 12,763 primary cancers occurred. Positive linear associations were observed between lifetime alcohol consumption and cancer-related mortality and total cancer incidence. J-shaped associations were observed between average lifetime alcohol consumption and overall mortality, cardiovascular-related mortality, and combined risk of death or cancer. In comparison to lifetime light alcohol drinkers (1–3 drinks per week), lifetime never or infrequent drinkers (<1 drink/week), as well as heavy (2–<3 drinks/day) and very heavy drinkers (3+ drinks/day) had increased overall mortality and combined risk of cancer or death. Corresponding hazard ratios (HRs) and 95% confidence intervals (CIs) for combined risk of cancer or death, respectively, were 1.09 (1.01–1.13) for never drinkers, 1.08 (1.03–1.13) for infrequent drinkers, 1.10 (1.02–1.18) for heavy drinkers, and 1.21 (1.13–1.30) for very heavy drinkers. This analysis is limited to older adults, and residual confounding by socioeconomic factors is possible.

**Conclusions:**

The study supports a J-shaped association between alcohol and mortality in older adults, which remains after adjustment for cancer risk. The results indicate that intakes below 1 drink per day were associated with the lowest risk of death.

**Trial registration:**

NCT00339495 (ClinicalTrials.gov).

## Abstract 2

In this cohort study, Andrew Kunzmann and colleagues examine whether risk of cancer or death differed in older adults with different lifetime alcohol intake.

## Author summary

**Why was this study done?:**

- The detrimental health impacts of heavy alcohol intakes are well known, and even light-moderate alcohol intakes have been linked to increased risks of cancer.
- However, light-moderate drinking has also been suggested to be protective for cardiovascular disease, which has led to contradictory public health messages. We conducted this study to further understand the complex relationship between light-moderate alcohol consumption, cancer, and mortality.

**What did the researchers do and find?:**

- We analysed whether risk of cancer or death differed in individuals with different lifetime alcohol intakes, using data from approximately 100,000 individuals in the United States.
- The results suggested that risk of some cancers increased with each additional alcoholic drink per week consumed.
- However, combined risk of cancer or death was lowest in light drinkers consuming less than 1 drink per day, rather than drinkers with higher intakes.

**What do these findings mean?:**

- These results could help inform future US guidelines, which currently recommend less than 2 drinks per day for men and less than 1 drink per day for women.
- This evidence should not be taken to support a protective effect of light drinking.

## Introduction

Alcohol consumption appears to have a complex and somewhat controversial relationship to health [1–4]. The “J-shaped” relationship between alcohol intake and mortality, particularly from cardiovascular disease, observed in various cohort studies has been well cited within both scientific and mainstream publications [5–8]. However, concerns over the quality of the evidence supporting the J-shaped association [1,2], combined with alcohol’s role as a risk factor for a range of cancers [9,10], has led to conflicting messages surrounding the health implications of light to moderate alcohol consumption.

Criticisms over the quality of evidence supporting a J-shaped association with mortality often surround the methods used to assess and categorise alcohol intakes [2,11]. Many of the previous cohort studies assessing the role of alcohol in mortality and cancer have focussed on alcohol intakes measured at baseline, with former drinkers excluded from current drinker categories [11]. However, this approach may lead to an underestimation of the negative health effects of drinking alcohol, as former drinkers may have stopped drinking due to adverse health effects or health scares related to their drinking habits [1,2]. Measurement of alcohol intakes over an individual’s lifetime and classification according to average lifetime alcohol intakes helps to avoid the bias that occurs when separating former drinkers from current drinkers and gives a more comprehensive assessment of an individual’s drinking habits.

To date, few studies [5,6,12] have evaluated average lifetime alcohol consumption and mortality [13], which could help clarify the association. No studies of lifetime alcohol intake, to our knowledge, have examined the impact on mortality and cancer risk simultaneously, to allow a direct comparison of alcohol’s association with both important health outcomes.

A more comprehensive overview of alcohol and risk of cancer and death outcomes would also help to provide clearer guidelines on alcohol consumption. The 2015–2020 U.S. Dietary Guidelines for Americans currently recommend less than 1 drink per day in women and less than 2 drinks per day in men [14].

In this study, we aimed to simultaneously examine the association between average lifetime alcohol intake and mortality as well as cancer risk in a large cohort with prospective follow-up data. We also conducted a novel analysis of combined risk of cancer incidence or death, to account for differences in timing and absolute risk of these outcomes.

## Methods

### Study population

The Prostate, Lung, Colorectal, and Ovarian (PLCO) Cancer Screening Trial is a randomised trial designed to evaluate the impact of screening modalities on cancer mortality, as described previously [15]. Briefly, 154,952 individuals, aged 55–74 years, were recruited via 10 centres in the US (Birmingham, Alabama; Boulder, Colorado; Detroit, Michigan; Honolulu, Hawaii; Los Angeles, California; Minneapolis, Minnesota; Pittsburgh, Pennsylvania; Salt Lake City, Utah; St. Louis, Missouri; Marshfield, Wisconsin; Washington, DC) between 1993 and 2001. All participants provided written informed consent and the study was approved by the Institutional Review Boards at the National Cancer Institute and the 10 recruitment centres. This study is reported as per the Strengthening the Reporting of Observational Studies in Epidemiology (STROBE) guidelines (S1 Checklist).

### Assessment of demographic and lifestyle variables

At study entry, participants self-completed a baseline questionnaire, which enquired information on demographic variables, including age, gender, race/ethnicity, marital status, educational attainment, personal and family medical history, tobacco smoking habits (including duration and frequency), medication use, and anthropometry.

### Alcohol use assessment

Alcohol consumption was assessed using the validated PLCO Diet History Questionnaire (DHQ) [16], which was introduced into the trial in December 1998 and was completed by approximately 77% of all participants in both arms of the trial. Participants recruited after 1998 completed the DHQ at baseline for the nonscreening arm and approximately 3 years after baseline for the screening arm, whereas previously recruited participants were invited to complete the DHQ in 1999 or 2000.

The DHQ assessed historical drinking by inquiring about the amount of beer (12 ounce bottles or cans: 1 U.S. Department of Agriculture My Pyramid cup equivalent [17]), wine (5 ounce glasses: 1 cup equivalent), and liquor (1.5 ounce shots, including mixed drinks: 1 cup equivalent) the participant consumed per week between the ages of 18 and 24, 25 and 39, 40 and 54 years old, and at 55+ years, respectively. The DHQ also inquired about the amount and frequency the participant drank beer, wine (or wine coolers), and liquor (including liquor in mixed drinks) over the previous summer, as well as the rest of the previous year, to account for seasonal differences. The frequency was converted into days and an average daily alcohol intake of each type of alcohol was calculated as follows (using midpoints where necessary):

(amount*frequencyinsummer+(amount*frequencyfortherestoftheyear*3))/4

### Categories of average lifetime alcohol intake

Average lifetime alcohol intake was calculated as a weighted average by multiplying the daily alcohol intake by the number of years in each age category and adding the categories together (e.g., intakes at 18–24 accounted for 7 years and intakes in the year prior to DHQ accounted for 1 year). Never drinkers were defined as having reported no alcohol consumption at any age. For some analyses, categories of drinking (infrequent drinkers [>0–<1 drinks/week], light drinkers [1–<3 drinks/week], somewhat light drinkers [3–<5 drinks/week], light-moderate drinkers [5–<7 drinks/week], moderate drinkers [1–<2 drinks/day], heavy drinkers [2–<3 drinks/day], very heavy drinkers [3+ drinks/day]) were chosen to be narrow enough to identify differences between groups and to allow relative comparability to previous studies [5], whilst being easily interpreted without limiting statistical power. The light drinker (1–<3 drinks/week) category was chosen as the reference category for mortality and combined outcomes, as these individuals were hypothesised to have the lowest mortality. The never drinker category was chosen as the reference category for risk of cancer, as it was hypothesised that these individuals would have the lowest cancer risk.

### Outcome assessment

Vital status during follow-up was ascertained by annual mailed questionnaires and periodic linkage to the National Death Index. We used International Classification of Diseases, 9th Revision (ICD-9), codes to classify the underlying cause of death specified on the death certificate. Causes of death, in order, were defined as follows: cardiovascular disease, including heart disease (ICD-9 codes 390–398, 402, 404, and 410–429) and stroke (ICD-9 codes 430–434 and 436–438); cancer (ICD-9 codes 140–239).

Cancer diagnoses were ascertained through assessment of medical records from follow-up investigations following screening by trained personnel, through annual questionnaires, and linkage to the National Death Index. All cancers were pathologically confirmed through medical record abstraction by trained personnel. Total cancer includes incident cancers diagnosed after completion of DHQ but prior to death, excluding nonmelanoma skin cancer. Alcohol-related cancers include breast cancer, colorectal cancer, head and neck cancer, liver cancer, and esophageal cancer [10,18].

The primary outcomes of interest were overall mortality (death from any cause), mortality from cardiovascular disease and cancer, as well as total cancer risk (incidence of any cancer, excluding nonmelanoma skin cancer) and a novel analysis, which classed either incidence of any cancer (excluding nonmelanoma skin cancer) or death from any cause as the event of interest. Deaths from homicides, suicide or accidents, and other causes of death were reported as secondary outcomes. Follow-up time was calculated from the date of DHQ completion to the first of either death, 13 years after randomisation or 31 December 2009, or date of first incidence of any cancer (excluding nonmelanoma skin cancer) in analyses including cancer as an outcome.

### Cohort selection

**Fig 1:**
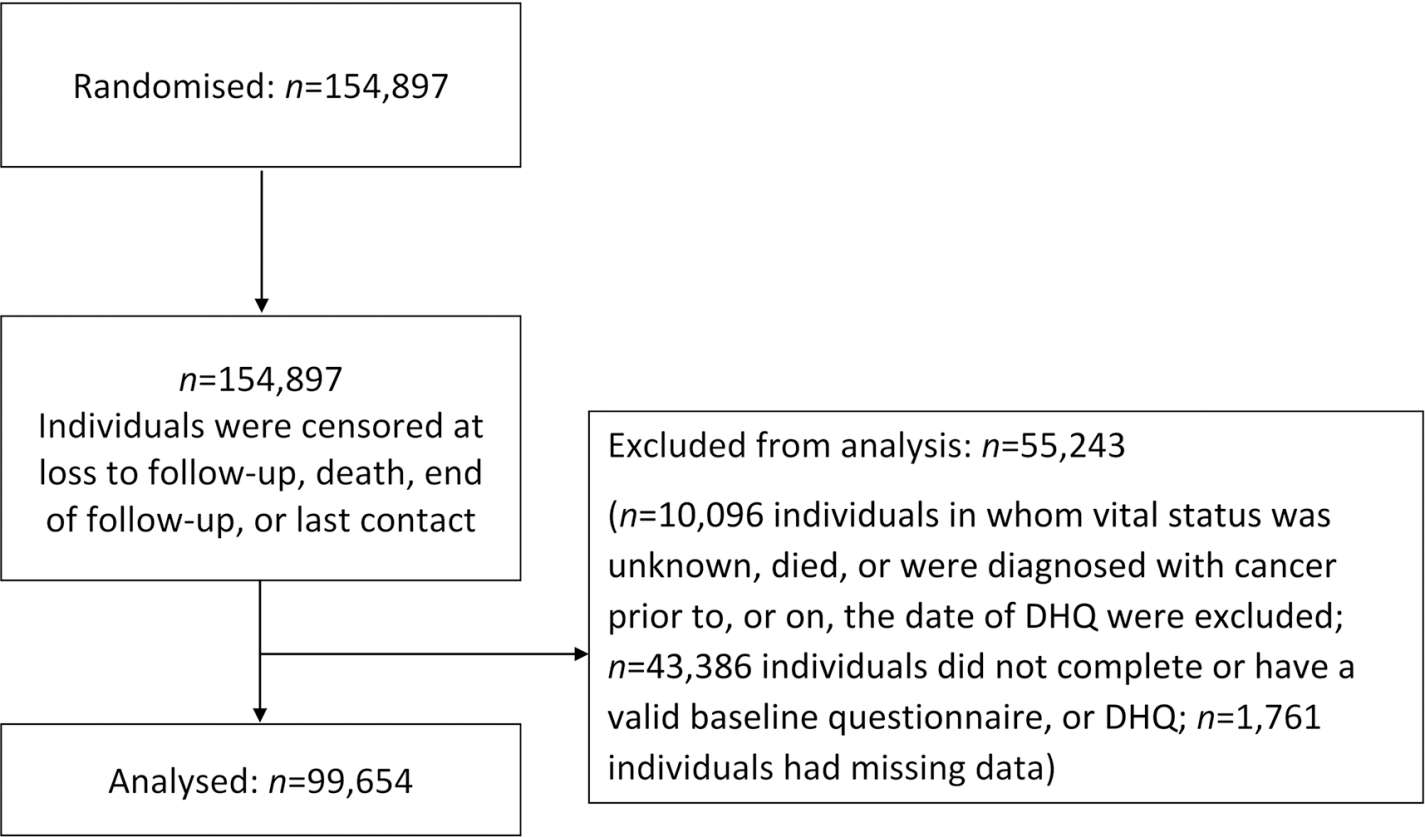
STROBE flow diagram of participant inclusion.

A total of 154,952 individuals agreed to participate in the trial. Individuals who died or were diagnosed with cancer prior to or on the date of DHQ were excluded (*n* = 10,096). Individuals who did not complete the baseline questionnaire (*n* = 4,920) or the DHQ (*n* = 33,245), did not have valid DHQ (*n* = 5,221), or had missing information for alcohol or key confounders (*n* = 1,761) were excluded. After exclusions, 99,654 individuals were eligible for analysis, of whom 9,599 died from any cause and 12,763 were diagnosed with any cancer (excluding nonmelanoma skin cancer). A STROBE flowchart of participant selection is included in Fig 1.

### Statistical analysis

Chi-squared tests (categorical variables) and ANOVA tests (continuous variables) were used to assess the association between baseline characteristics and average lifetime alcohol intakes. Cox proportional hazards models were used to estimate the hazard ratios (HRs) and corresponding 95% confidence intervals (CIs) for the association between average lifetime alcohol intake and each outcome (all-cause mortality, cardiovascular-related mortality, cancer-related mortality, and cancer incidence, including total and alcohol-related cancers), in men and women separately. Age was used as the underlying timescale in all analyses [19]. Random effects meta-analyses using the inverse variance as the weight were used to provide a combined estimate across men and women for each level of intake.

Adjusted models included race/ethnicity (non-Hispanic white, non-Hispanic black, Asian, Hispanic/Other), study centre, randomisation group (control group or screening group), body mass index (BMI), year of DHQ completion, smoking status by pack-years (never smokers, former smoker reporting ≤25 pack-years, former smoker reporting ≥25 pack-years, current reporting ≤25 pack-years, current reporting ≥25 pack years, cigar/pipe only smokers), marital status, education, family history of cancer (cancer outcomes only), intakes of total energy (kcal/day), calcium, red meat, processed meat, fiber, coffee, fruit and vegetables, and menopausal hormone replacement therapy use (HRT, in women only). No model selection for confounders was done, as recommended by the reviewer; all variables were included. At the reviewer’s suggestion, BMI, intakes of total energy, calcium, red meat, processed meat, fiber, coffee, fruit and vegetables were included as continuous variables instead of categorical variables.

Restricted cubic spline models [20] were fitted with varying number of knots to assess the dose-response trend in the association between average lifetime alcohol intake (as a continuous variable) and each outcome after full adjustment. Never drinkers were classified as the reference category for all restricted cubic spline analyses due to methodological constraints. Akaike's information criterion [21] and likelihood ratio tests were used to investigate if there was a significant improvement to the model fit when fitting restricted cubic spline models, with varying number of knots, compared to a linear model with average lifetime alcohol intake as a continuous variable. If the linear model provided the best fit for an outcome, Cox proportional hazards models were conducted, including average lifetime alcohol intakes as a continuous variable.

A priori stratified analyses were conducted by age (<65 versus 65+), randomisation group (control versus screening arm), BMI (>18.5–<25 versus >25 kg/m^2^), and smoking status (ever versus never) among the entire cohort (without gender stratification to ensure adequate statistical power) using restricted cubic splines for total mortality and total cancer risk. Secondary analyses reported results for primary outcomes and secondary outcomes from Cox proportional hazards models using alternative reference categories to those used in main analyses: never drinkers for mortality outcomes and light drinkers for cancer incidence outcomes. Further outcomes evaluated included mortality from accidents/injuries/homicide/suicide and mortality from any other cause, as well as risk of alcohol-related cancers, other cancers, and individual types of cancer (including all alcohol-related cancers and PLCO cancers). Post hoc sensitivity analyses based on suggestions from reviewers also assessed overall mortality in current and former drinkers relative to never drinkers and the association between lifetime alcohol consumption with and death from any cause within cancer patients.

Restricted cubic splines were also used to assess the association between different types of alcohol (beer, wine, and liquor) and overall mortality and cancer incidence (accounting for intakes of other types of alcohol). A 2-tailed *p-*value < 0.05 was considered significant and analyses were conducted using Stata/SE statistical software (version 14.0, College Station, TX, USA). A copy of the research proposal is included in the supplementary material (S1 Text).

## Results

### Participant characteristics

Our analysis included 99,654 individuals, including 51,306 women (68.7%) and 48,348 men (31.3%). The median follow-up time was 8.9 years, with 9,599 deaths and 12,763 primary cancers recorded over a total 836,740 person-years of follow-up. Drinking patterns differed between men and women, with the proportion of women compared to men higher amongst never and infrequent drinkers and lower amongst moderate, heavy, and very heavy drinkers (Table 1). The average median lifetime alcohol intake was 1.78 drinks per week (0.25 per day), with men reporting higher intakes (4.02 drinks/week) than women (0.80 drinks per week).

**Table 1:** Baseline characteristics of men and women in the study population according to average lifetime alcohol intake (MPED equivalent drinks).

	Never drinkers	Infrequent (0–<1/week)	Light (1–<3/week)	Somewhat light (3–<5/week)	Light-moderate (5–<7/week)	Moderate (1–<2/day)	Heavy (2–<3/day)	Very heavy (3+/day)	Total
**Men**									
Number of participants (%^1^)	3,812 (31.3)	7,584 (27.6)	9,155 (43.3)	6,851 (57.6)	4,982 (67.3)	7,984 (75.6)	3,955 (85.6)	4,025 (91.6)	48,348 (48.5)
Age at DHQ (SD)	67 (8)	66 (10)	66 (9)	65 (9)	65 (9)	65 (9)	65 (8)	64 (9)	65 (10)
Marital status (married, %)	3,429 (90)	6,606 (87.1)	7,912 (86.4)	5,941 (86.7)	4,293 (86.2)	6,808 (85.3)	3,251 (82.2)	3,002 (74.6)	41,242 (85.3)
Race (non-Hispanic white, %)	3,328 (87.3)	6,683 (88.1)	8,388 (91.6)	6,268 (91.5)	4,573 (91.8)	7,393 (92.6)	3,660 (92.5)	3,588 (89.1)	43,881 (90.8)
Education (University degree, %)	1,809 (47.5)	3,334 (44)	4,090 (44.7)	3,038 (44.3)	2,101 (42.2)	3,475 (43.5)	1,452 (36.7)	1,064 (26.4)	20,363 (42.1)
Family history of cancer (%)	1,948 (51.1)	3,910 (51.6)	4,741 (51.8)	3,611 (52.7)	2,681 (53.8)	4,181 (52.4)	2,107 (53.3)	2,074 (51.5)	25,253 (52.2)
Body mass index (30+, %)	858 (22.5)	1,717 (22.6)	2,028 (22.2)	1,566 (22.9)	1,116 (22.4)	1,842 (23.1)	943 (23.8)	1,118 (27.8)	11,188 (23.1)
Current smoker (>25 pack-years, %)	109 (2.9)	407 (5.4)	625 (6.8)	531 (7.8)	431 (8.7)	806 (10.1)	542 (13.7)	773 (19.2)	4,224 (8.7)
Coffee intake (>3 cups/day, %)	259 (6.8)	896 (11.8)	1,464 (16)	1,162 (17)	950 (19.1)	1,708 (21.4)	957 (24.2)	1,155 (28.7)	8,551 (17.7)
Energy intake (kcal, SD)	1,708 (941)	1,703 (927)	1,739 (891)	1,805 (906)	1,898 (938)	1,987 (963)	2,102 (1031)	2,253 (1303)	1,418 (700)
Processed meat intake (g/1,000 kcal, SD)	7.3 (9.9)	8.3 (10.4)	8.8 (10.2)	9 (9.9)	9 (9.8)	9.1 (9.8)	8.7 (9.8)	8.8 (10.1)	5.5 (7.1)
Red meat intake (MPED/1,000 kcal, SD)	23.5 (20.7)	25.2 (21.8)	26.4 (21.7)	28.2 (22.4)	28.7 (22.3)	29.3 (23)	28.9 (23)	28.4 (23.9)	21.5 (18.9)
Fruit and vegetable intake (MPED/1,000 kcal, SD)	2.1 (1.3)	2 (1.2)	1.9 (1.1)	1.8 (1)	1.8 (1)	1.7 (1)	1.6 (1)	1.5 (1)	2.4 (1.3)
Dietary fibre intake (g/1,000 kcal, SD)	10.3 (4.5)	10 (4.4)	9.7 (4.1)	9.4 (3.9)	9.1 (3.8)	8.8 (3.7)	8.4 (3.8)	7.8 (4)	11 (4.5)
Calcium intake (mg/1,000 kcal, SD)	469.2 (298.5)	444.5 (277.9)	437.2 (257.6)	424.8 (247.5)	410 (232.4)	397.8 (220.7)	381.2 (220.8)	366.9 (215.1)	706.5 (539.4)
**Women**									
Number of participants (%^1^)	8,368 (68.7)	19,869 (72.4)	12,004 (56.7)	5,040 (42.4)	2,418 (32.7)	2,573 (24.4)	664 (14.4)	370 (8.4)	51,306 (51.5)
Age at DHQ (SD)	66 (9)	65 (10)	64 (9)	64 (9)	65 (8)	64 (9)	64 (9)	63 (8)	65 (10)
Marital status (married, %)	6,381 (76.3)	14,313 (72)	8,650 (72.1)	3,578 (71)	1,707 (70.6)	1,711 (66.5)	411 (61.9)	173 (46.8)	36,924 (72)
Race (non-Hispanic white, %)	7,181 (85.8)	17,900 (90.1)	11,329 (94.4)	4,780 (94.8)	2,273 (94)	2,429 (94.4)	624 (94)	323 (87.3)	46,839 (91.3)
Education (University degree, %)	2,135 (25.5)	5,725 (28.8)	3,993 (33.3)	1,795 (35.6)	874 (36.1)	965 (37.5)	230 (34.6)	101 (27.3)	15,818 (30.8)
Family history of cancer (%)	5,097 (60.9)	11,922 (60)	7,037 (58.6)	2,995 (59.4)	1,411 (58.4)	1,517 (59)	401 (60.4)	213 (57.6)	30,593 (59.6)
Body mass index (30+, %)	2,328 (27.8)	5,376 (27.1)	2,556 (21.3)	937 (18.6)	459 (19)	477 (18.5)	131 (19.7)	105 (28.4)	12,369 (24.1)
Current smoker (>25 pack-years, %)	147 (1.8)	1,043 (5.2)	821 (6.8)	405 (8)	248 (10.3)	318 (12.4)	111 (16.7)	82 (22.2)	3,175 (6.2)
Coffee intake (>3 cups/day, %)	439 (5.2)	2,140 (10.8)	1,607 (13.4)	769 (15.3)	376 (15.6)	430 (16.7)	136 (20.5)	81 (21.9)	5,978 (11.7)
Energy intake (kcal, SD)	1,376 (713)	1,363 (688)	1,421 (671)	1,479 (687)	1,545 (684)	1,620 (718)	1,641 (765)	1,718 (907)	1,418 (700)
Processed meat intake (g/1,000 kcal, SD)	4.8 (7)	5.6 (7.2)	5.7 (7.1)	5.5 (7)	5.6 (6.8)	5.4 (7.2)	5.5 (6.7)	5.3 (7.2)	5.5 (7.1)
Red meat intake (MPED/1,000 kcal, SD)	19.3 (18.5)	21.3 (19.1)	22.5 (18.9)	22.4 (18.3)	22.3 (19.1)	22.4 (19.1)	22.4 (18.5)	20.3 (19.3)	21.5 (18.9)
Fruit & vegetable intake (MPED/1,000 kcal, SD)	2.6 (1.5)	2.5 (1.4)	2.4 (1.2)	2.3 (1.2)	2.2 (1.2)	2.1 (1.2)	2.1 (1.3)	1.9 (1.3)	2.4 (1.3)
Dietary fibre intake (g/1,000 kcal, SD)	11.5 (4.9)	11.3 (4.6)	10.9 (4.1)	10.4 (4)	10 (4.1)	9.7 (4.1)	9.2 (4.3)	9.2 (5.1)	11 (4.5)
Calcium intake (mg/1,000 kcal, SD)	722.6 (566)	708.1 (557.2)	719.3 (534.6)	700.5 (501.2)	675.1 (493)	662.1 (490.7)	634.3 (465.8)	611.5 (495)	706.5 (539.4)
Hormone replacement therapy (current, %)	4,074 (48.7)	10,157 (51.1)	6,536 (54.4)	2,861 (56.8)	1,344 (55.6)	1,435 (55.8)	354 (53.3)	183 (49.5)	26,944 (52.5)

^1^Percentage of all participants, all others are sex-specific percentages.

Abbreviations: DHQ, Diet History Questionnaire; kcal, kilocalorie; MPED, My Pyramid equivalent; SD, standard deviation.

In men, heavier drinkers were less likely to be married or have completed a university degree; more likely to be smokers or obese; and tended to have lower intakes of fruits and vegetables, dietary fibre, and total calcium. Similar patterns were apparent in women, although never and infrequent drinkers tended to be more similar to very heavy drinkers (3+ drinks per day) than light-moderate drinkers in terms of university education and obesity. HRT use was least common amongst never drinkers (Table 1).

### Average lifetime alcohol consumption and mortality

#### Total mortality

**Fig 2:**
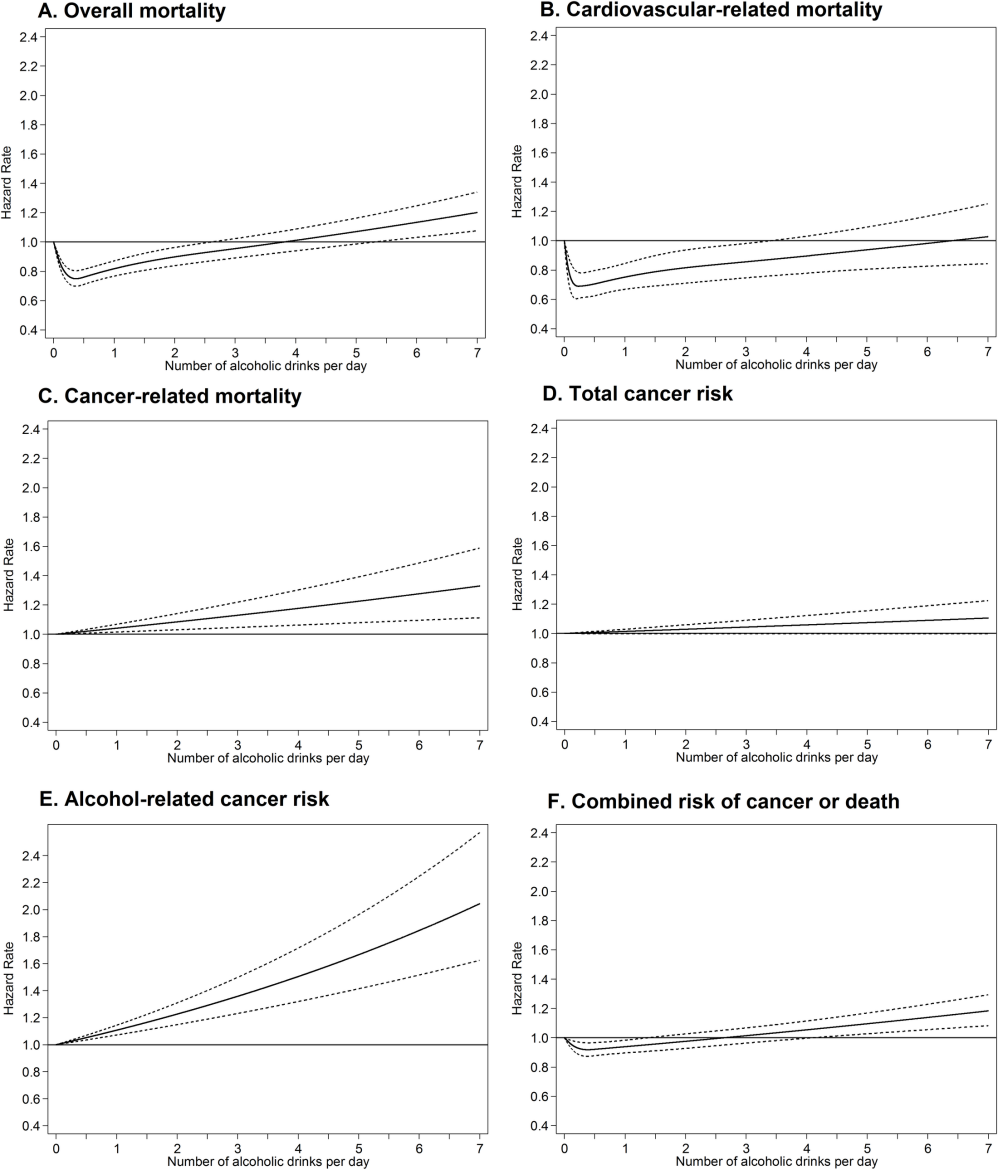
Restricted cubic splines for the association between average lifetime alcohol intake and overall mortality (A, 4 knots), cardiovascular-related mortality (5 knots), cancer-related mortality (C, linear), total cancer risk (D, linear), risk of alcohol-related cancers (E, linear), and risk of cancer or death (F, 4 knots/3 splines) in men and women combined. Results are adjusted for gender, study centre, race, BMI, randomisation group, smoking status, year of DHQ completion, marital status, educational attainment, family history of cancer, HRT use (women only), energy intake, red meat intakes per 1,000 kcal, processed meat intakes per 1,000 kcal, coffee intake, fruit and vegetable intake, fibre intake, and calcium intake). Akaike's information criterion and likelihood ratio tests were used to investigate if there was a significant improvement to the model fit when fitting restricted cubic spline models, with varying number of knots, compared to a linear model with average lifetime alcohol intake as a continuous variable. BMI, body mass index; DHQ, Diet History Questionnaire; HRT, hormone replacement therapy.

Restricted cubic splines analyses indicated J-shaped relationships between average lifetime alcohol intakes and risk of overall mortality, with risk of death lowest amongst those whose intakes were below 0.5 drinks per day, with increasing risk at higher intakes (Fig 2A, S1 Fig).

**Fig 3:**
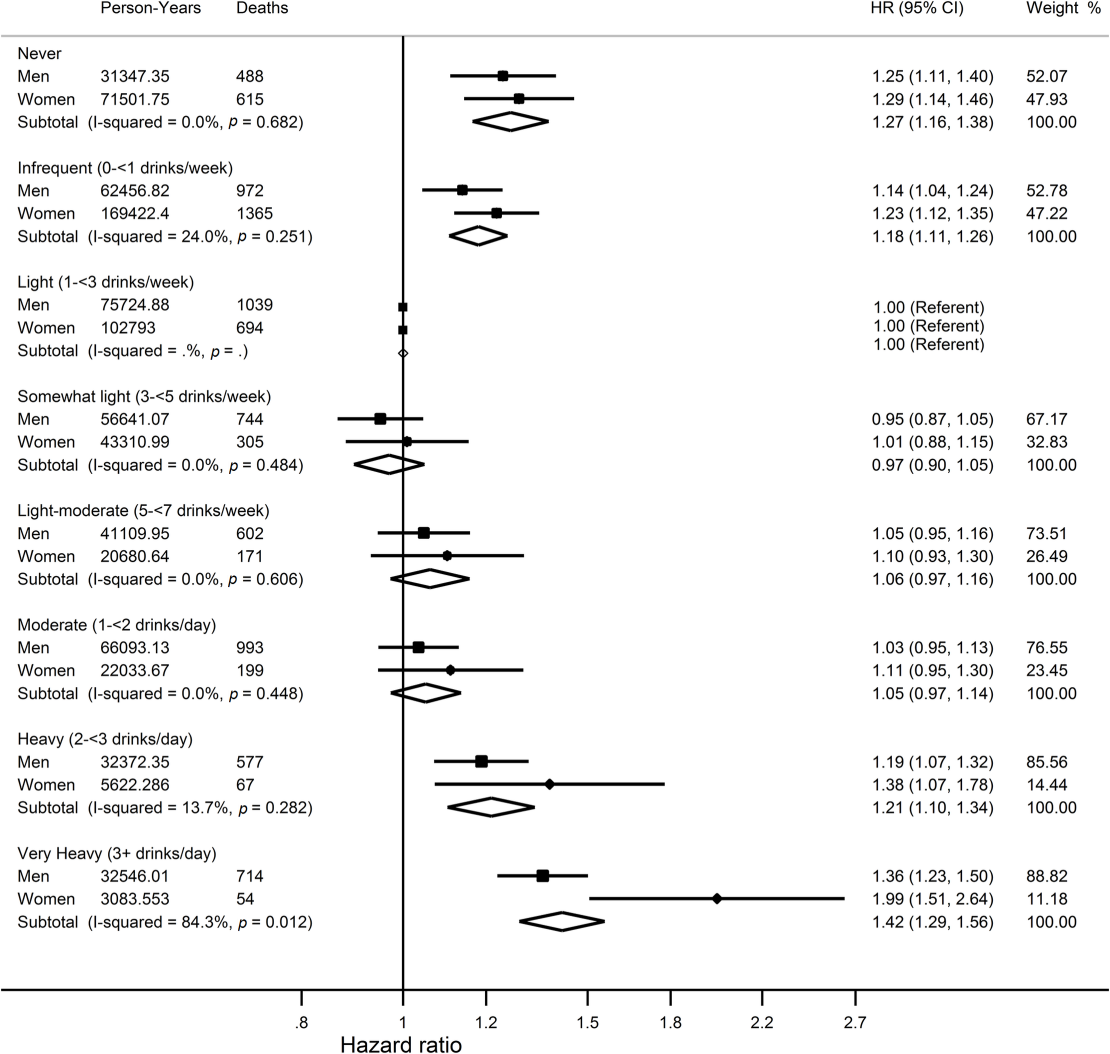
Hazard ratios and 95% confidence intervals for the association between categories of average lifetime alcohol intake and overall mortality compared to light drinkers who reported drinking 1–3 alcoholic drinks per week. Results are adjusted for study centre, race, BMI, randomisation group, smoking status, year of DHQ completion, marital status, educational attainment, family history of cancer (cancer mortality only), hormone replacement therapy use (women only), energy intake, red meat intakes per 1,000 kcal, processed meat intakes per 1,000 kcal, coffee intake, fruit and vegetable intake, fibre intake, and calcium intake. BMI, body mass index; DHQ, Diet History Questionnaire.

In men and women, in comparison to light drinkers (1–3 drinks per week), lifetime never drinkers, infrequent drinkers (<1 drink/week) as well as heavy drinkers (2–<3 drinks/day), and very heavy drinkers (3+ drinks/day) had an increased risk of mortality (Fig 3). The corresponding HRs (and 95% CI) among men and women, respectively, were 1.25 (1.11–1.40) and 1.29 (1.14–1.46) for never drinkers, 1.14 (1.04–1.24) and 1.23 (1.12–1.35) for infrequent drinkers, 1.19 (1.07–1.32) and 1.38 (1.07–1.78) for heavy drinkers, and 1.36 (1.23–1.50) and 1.99 (1.51–2.64) for very heavy drinkers (Fig 3). No major differences were observed by age, smoking status, BMI, or randomisation group (S2 Fig).

When assessing intakes in the year prior to DHQ, former drinkers (HR 1.12, 95% CI 1.04–1.22) and heavy current drinkers (>2 drinks per day, HR 1.21, 95% CI 1.03–1.42) were at an increased risk of death compared to never drinkers. Light-moderate drinkers were at a reduced risk of death (>0–<2 drinks per day, HR 0.80, 95% CI 0.74–0.86).

#### Cause-specific mortality

Analyses of specific causes of mortality also revealed J-shaped associations for cardiovascular-related mortality (Fig 2B) but a linear association for cancer-related mortality (Fig 2C). Cardiovascular-related mortality was increased in lifetime never drinkers (HR 1.58, 95% CI 1.26–1.98) and infrequent drinkers (HR 1.38, 95% CI 1.15–1.65) in women, and very heavy drinkers (HR 1.24, 95% CI 1.05–1.47) in men compared to lifetime light drinkers (S1 Table and S2 Table). Cancer-related mortality was increased in heavy drinkers in both men (HR 1.24, 95% CI 1.04–1.48) and women (HR 1.52, 95% CI 1.06–2.18) and very heavy drinkers in men (HR 1.23, 95% CI 1.03–1.47) compared to lifetime light drinkers; especially for mortality from alcohol-related cancers (S1 Table and S2 Table). Similar results were apparent when assessing deaths from any cause within cancer patients. Among men diagnosed with cancer, higher risks of death were observed for never drinkers (HR 1.20, 95% CI 1.00–1.46) and very heavy drinkers (HR 1.27, 95% CI 1.08–1.50) relative to light drinkers. A J-shaped association was apparent for mortality from other nonviolent causes but not for mortality due to homicide, suicide, or accidents (S1 Table and S2 Table).

### Average lifetime alcohol and cancer incidence

**Fig 4:**
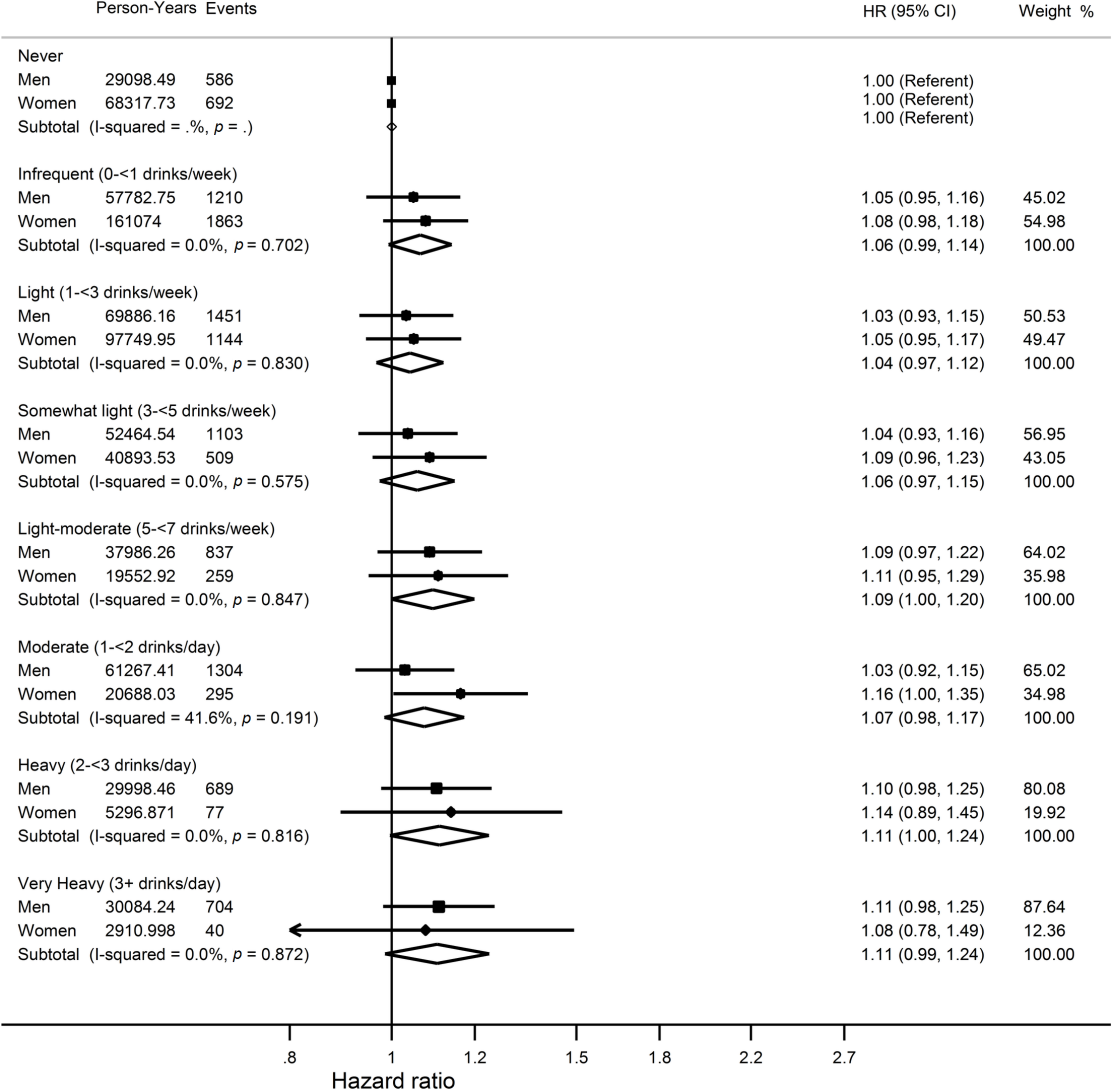
Hazard ratios and 95% confidence intervals for the association between categories of average lifetime alcohol intake and total cancer risk compared to never drinkers. Results are adjusted for study centre, race, BMI, randomisation group, smoking status, year of DHQ completion, marital status, educational attainment, family history of cancer (risk of cancer or death only), HRT use (women only), energy intake, red meat intakes per 1,000 kcal, processed meat intakes per 1,000 kcal, coffee intake, fruit and vegetable intake, fibre intake, and calcium intake. BMI, body mass index; DHQ, Diet History Questionnaire; HRT, hormone replacement therapy.

Analyses indicated that average lifetime alcohol intakes were linearly associated with total risk of cancer (Fig 2D) and risk of ‘alcohol-related’ cancers (Fig 2E). Light-moderate drinkers (HR 1.09, 95% CI 1.00–1.20) and heavy drinkers (HR 1.11, 95% CI 1.00–1.24) were at an increased risk of cancer overall (when combining men and women) when compared to never drinkers (Fig 4). When average lifetime alcohol was assessed as a continuous variable, each additional drink per day was associated with a small increase in total risk of cancer in men (HR 1.01, 95% CI 1.00–1.02). No major differences were observed by age, smoking status, BMI, or randomisation group (S3 Fig), but differences by type of alcohol were observed (S4 Fig). Risk of ‘alcohol-related’ cancer was 66% higher in very heavy drinkers than in never drinkers in men (HR 1.66, 95% CI 1.16–2.38). Each additional drink per day was associated with an increased risk of ‘alcohol-related’ cancers in men (HR 1.06, 95% CI 1.04–1.08). Analyses by type of cancer revealed very heavy drinking was associated with increased risk of head and neck cancers (HR 3.12, 95% CI 1.54–6.34), liver cancer (HR 3.53, 95% CI 1.26–9.87), and esophageal cancer (HR 3.99, 95% CI 1.47–10.82) (S3 Table).

### Average lifetime alcohol intake and combined risk of cancer or death

**Fig 5:**
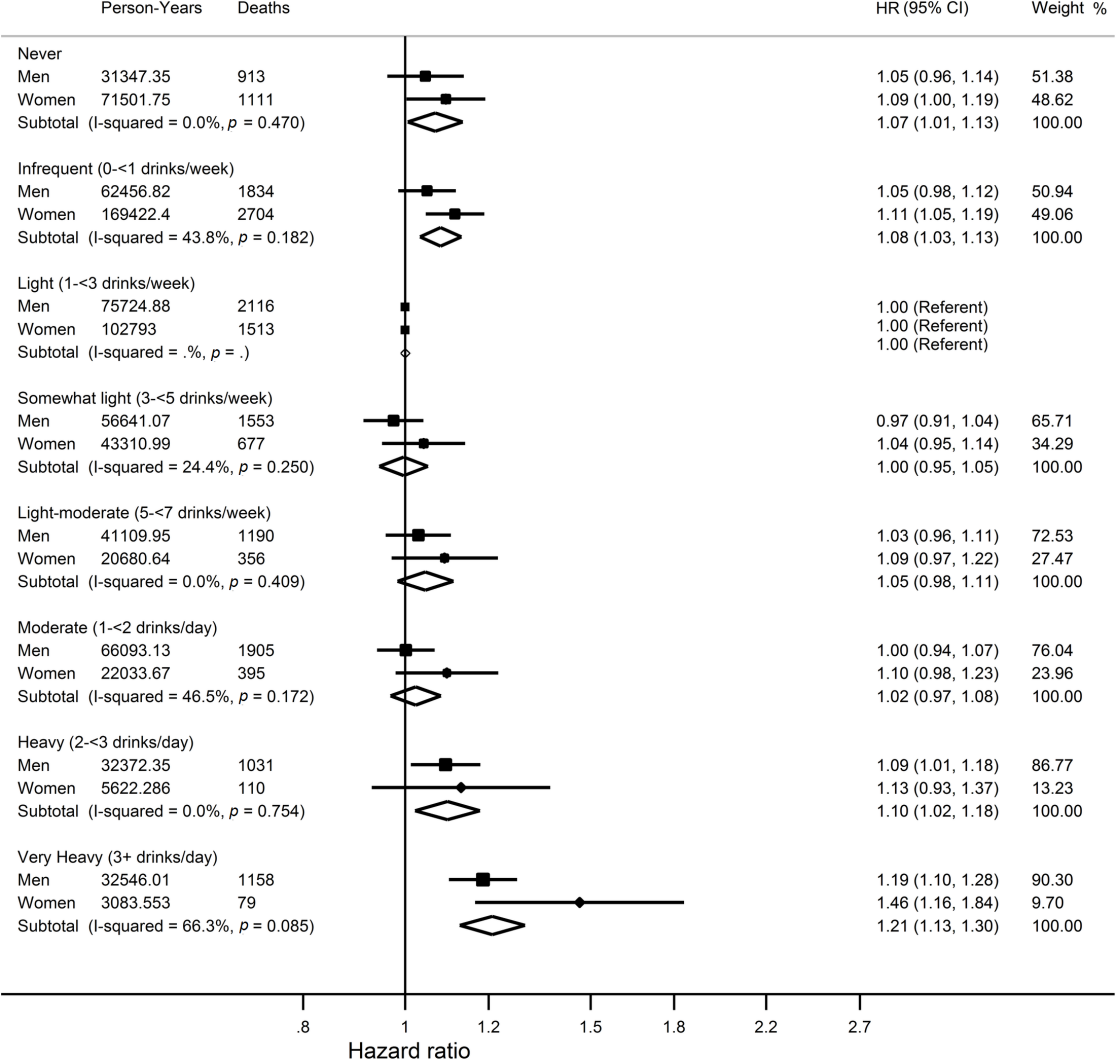
Hazard ratios and 95% confidence intervals for the association between categories of average lifetime alcohol intake and combined risk of cancer or death compared to light drinkers who reported drinking 1–3 alcoholic drinks per week. Results are adjusted for study centre, race, BMI, randomisation group, smoking status, year of DHQ completion, marital status, educational attainment, family history of cancer (cancer mortality only), hormone replacement therapy use (women only), energy intake, red meat intakes per 1,000 kcal, processed meat intakes per 1,000 kcal, coffee intake, fruit and vegetable intake, fibre intake, and calcium intake. BMI, body mass index; DHQ, Diet History Questionnaire.

A J-shaped relationship was apparent between average lifetime alcohol intakes and combined risk of cancer or death, with the lowest risk observed at intakes between 1 and 5 drinks per week (Fig 2F, S1 Fig). Never, infrequent, moderate, and very heavy drinkers had a higher combined risk of cancer or death than light drinkers (1–<3 drinks/week) overall (men and women combined) with HRs of 1.07 (95% CI 1.01–1.13) for never drinkers, 1.08 (95% CI 1.03–1.13) for infrequent drinkers, 1.10 (95% CI 1.02–1.18) for heavy drinkers, and 1.21 (95% CI 1.13–1.30) for very heavy drinkers. Results were similar for women alone, whereas in men the differences were only statistically significant for heavy (HR 1.09, 95% CI 1.01–1.18) and very heavy drinkers (HR 1.19, 95% CI 1.10–1.28) (Fig 5).

## Discussion

In this analysis, we observed nonlinear, “J-shaped” associations between average lifetime alcohol consumption and overall and cardiovascular-related mortality. Light lifetime alcohol consumption was associated with reduced overall and cardiovascular-related mortality compared to never drinking. Higher average lifetime alcohol consumption was also linearly associated with increased cancer-related mortality and cancer incidence. Combining the risk of cancer and death into a single analysis attenuated the modest reduction in risk observed for light drinkers, suggesting only a small benefit in risk for light drinkers compared to never drinkers.

Whilst existing research is largely consistent as to the harms of heavy drinking in terms of both cancer incidence and overall mortality, there are disparate messages regarding the safety of light to moderate alcohol consumption. Some argue that there is ‘no safe limit of alcohol’, largely on the basis of increased cancer incidence [22,23], and others emphasise the potential benefits for reducing cardiovascular mortality [3,24]. The results for lifetime alcohol consumption when mortality and cancer incidence were assessed separately in the current study were similarly disparate. The analysis of combined risk of death and cancer incidence aimed to account for these disparate associations. The results indicate that the J-shaped association and risk reductions observed for light alcohol intakes remained, albeit slightly attenuated, with small reductions in combined risk at light intakes (between 1 and <5 drinks per week). This study adds to the limited evidence base for women [13] and indicates the lowest combined risk of death and cancer incidence is at lighter intakes in both men and women. These results could help inform future US guidelines, which currently recommend less than 2 drinks per day for men and less than 1 drink per day for women [14]. The United Kingdom recently lowered their guidelines for men to recommend less than 14 units (about 1 drink) per week, several alcohol-free days per week, and limiting the total amount of alcohol in one session [23]. Mendelian randomisation studies indicating beneficial effects of alleles associated with reduced alcohol intakes on cardiovascular outcomes are consistent with our findings and could also inform these debates [25]. Further studies incorporating other major sources of morbidity, such as dementia [4,26], may better inform public health guidelines on the health impact of light drinking.

This large cohort study had a number of strengths and limitations. Previous studies reporting a J-shaped association between lifetime alcohol consumption and mortality have been criticised for measuring alcohol consumption at a single time point and separating ‘unhealthy’ former drinkers from current drinkers [11,27]. By examining the use of lifetime alcohol consumption, we were able to limit this potential bias. The present study is also, to our knowledge, the first study with prospective follow-up to assess the association between lifetime alcohol consumption and overall cancer risk.

Other studies have been criticised for having broader categories of consumption and advocating a reduction in mortality at implausibly low intakes [11]. In our study, we included a separate category for infrequent lifetime drinkers (>0–<1) to examine infrequent drinking more closely. The increased mortality in infrequent drinkers compared to light drinkers in our study suggests that these groups are indeed different and could explain why a systematic review found no reduction in mortality amongst ‘low volume drinkers’, which includes both infrequent and moderate drinkers (up to 2 drinks per day) [11]. The narrower classification of alcohol intakes used in the current study may have limited the statistical power to assess associations by cancer type.

The current study also had a larger proportion of never drinkers than a previous analysis of lifetime alcohol consumption from Europe [5]. This allowed for a more powerful assessment of the relative mortality between never drinkers and light drinkers. However, as an indirect consequence, the assessment of heavy drinking in women was limited by a smaller number of women reporting average lifetime alcohol consumption over 2 drinks per day in the current study.

Unlike many previous analyses, which focused on baseline or current consumption [24], this study assessed the risk associated with average lifetime alcohol consumption from the age of 18. However, persons were not enrolled in the study until after the age of 55 years and after DHQ completion. Thus, the study reflects mortality in older adults and the impact of alcohol consumption on mortality at younger ages could not be assessed, which could have been impacted by deaths from accidents, violence, or suicide [11,27], in particular. However, similar associations between alcohol and overall mortality have been seen previously in younger adults [24], which may alleviate concerns of an impact due to selection bias.

It is also possible that a certain degree of misclassification of alcohol consumption occurred due to social desirability bias or recall issues, particularly amongst individuals with infrequent alcohol consumption [28]. In addition, an assessment of episodic, binge drinking was not possible with the data collected in this study, although binge drinking is unlikely to exaggerate reductions in risk within light drinkers. We also did not have information on individuals who did not complete the DHQ, which also raises the potential for selection bias.

The role of socioeconomic status in health is also a potential source of confounding, as high socioeconomic status is associated with light-moderate alcohol intakes and better mortality outcomes [29,30]. Whilst we adjusted for educational attainment, smoking status, and various dietary factors that may be reflective of socioeconomic status, residual confounding is possible. Similarly, we were unable to adjust for physical activity, which could also be a source of residual confounding. Consistent with other screening trials, participants in the PLCO trial are more highly educated and less likely to smoke than the general population [31], suggesting that they are more homogenous than the US population at large. Thus, whilst the generalizability of findings may be weaker, the impact of such residual confounding is likely to be small. Finally, we were unable to evaluate cardiovascular disease incidence in this study, which would complement our analysis.

## Conclusions

The study supports a J-shaped association between alcohol and mortality in older adults, which remains after adjustment for cancer risk. The results indicate that intakes between 1 and <5 drinks per week were associated with the lowest combined risk of cancer or death. This study provides further insight into the complex relationship between alcohol consumption, cancer incidence, and disease mortality and may help inform public health guidelines.

## Supporting information

S1 Checklist STROBE checklist of items that should be included in reports of cohort studies. (DOCX)

S1 Text Study protocol when applying for use of the data. (DOCX)

S1 Fig A more detailed figure showing restricted cubic splines for the association between average lifetime alcohol intake up to 7 drinks per week and overall mortality (A, 4 knots) and risk of cancer or death (B, 4 knots/3 splines) in men and women combined (adjusted for gender, study centre, race, BMI, randomisation group, smoking status, year of DHQ completion, marital status, educational attainment, family history of cancer, HRT use [women only], energy intake, red meat intakes per 1,000 kcal, processed meat intakes per 1,000 kcal, coffee intake, fruit and vegetable intake, fibre intake, and calcium intake). BMI, body mass index; DHQ, Diet History Questionnaire; HRT, hormone replacement therapy.
(TIF)

S2 Fig HR (solid lines) and 95% CIs (dashed lines) for the association between average lifetime alcohol intake and overall mortality stratified by (A) age (individuals aged under 65: Black; over 65 years: Grey); (B) Smoking status (Never smokers: Black; Ever smokers: Grey); (C) BMI (Normal BMI: Black; BMI > 25: Grey); (D) Randomisation group (Control group: Black; Intervention group: Grey). BMI, body mass index; CI, confidence interval; HR, hazard ratio.
(TIF)

S3 Fig Hazard ratios (solid lines) and 95% confidence intervals (dashed lines) for the association between average lifetime alcohol intake and total cancer risk stratified by (A) age (individuals aged under 65: Black; over 65 years: Grey); (B) Smoking status (Never smokers: Black; Ever smokers: Grey); (C) BMI (Normal BMI: Black; BMI > 25: Grey); (D) Randomisation group (Control group: Black; Intervention group: Grey). BMI, body mass index.
(TIF)

S4 Fig Restricted cubic splines for the association (HR, solid lines) between average lifetime intakes of beer (yellow line), liquor (blue line), and wine (red line) and overall mortality (A, all 1 knots) and total cancer risk (B, wine: 1 knot; Beer and liquor: linear) in men and women combined (adjusted for gender, study centre, race, BMI, randomisation group, smoking status, year of DHQ completion, marital status, educational attainment, family history of cancer, HRT use [women only], energy intake, red meat intakes per 1,000 kcal, processed meat intakes per 1,000 kcal, coffee intake, fruit and vegetable intake, fibre intake, and calcium intake) with corresponding 95% confidence intervals (dashed lines). Akaike's information criterion and likelihood ratio tests were used to investigate if there was a significant improvement to the model fit when fitting restricted cubic spline models, with varying number of knots, compared to a linear model with average lifetime alcohol intake as a continuous variable. BMI, body mass index; DHQ, Diet History Questionnaire; HR, hazard ratio; HRT, hormone replacement therapy.
(TIF)

S1 Table The association between average lifetime alcohol and mortality using never drinkers as the reference category in men and women. (DOCX)

S2 Table The association between average lifetime alcohol and cancer outcomes using light drinkers as the reference category in men and women. (DOCX)

S3 Table The association between average lifetime alcohol intakes and risk of incident cancers (by type) using never drinkers as the reference category after adjustment for selected confounders. (DOCX)

## Abbreviations

BMI: body mass index
CI: confidence interval
DHQ: Diet History Questionnaire
HR: hazard ratio
HRT: hormone replacement therapy
ICD-9: International Classification of Diseases, 9th Revision
PLCO: Prostate, Lung, Colorectal, and Ovarian

## References

[1] Liang W, Chikritzhs T. The Association between Alcohol Exposure and Self-Reported Health Status: The Effect of Separating Former and Current Drinkers. PLoS ONE. 2013;8(2): 1–5. 10.1371/journal.pone.0055881 PMC3566043 23405228

[2] Chikritzhs T, Stockwell T, Naimi T, Andreasson S, Dangardt F, Liang W. Has the leaning tower of presumed health benefits from “moderate” alcohol use finally collapsed? Addiction. 2015;110(5): 726–727. doi: 10.1111/add.12828 25613200 10.1111/add.12828

[3] Bell S, Daskalopoulou M, Rapsomaniki E, George J, Britton A, Bobak M, et al Association between clinically recorded alcohol consumption and initial presentation of 12 cardiovascular diseases: population based cohort study using linked health records. BMJ. 2017;356: j909 doi: 10.1136/bmj.j909 28331015 10.1136/bmj.j909 PMC5594422

[4] Topiwala A, Allan CL, Valkanova V, Zsoldos E, Filippini N, Sexton C, et al Moderate alcohol consumption as risk factor for adverse brain outcomes and cognitive decline: longitudinal cohort study. BMJ. 2017;357: j2353 doi: 10.1136/bmj.j2353 28588063 10.1136/bmj.j2353 PMC5460586

[5] Ferrari P, Licaj I, Muller DC, Kragh Andersen P, Johansson M, Boeing H, et al Lifetime alcohol use and overall and cause-specific mortality in the European Prospective Investigation into Cancer and nutrition (EPIC) study. BMJ Open. 2014;4(7): e005245 doi: 10.1136/bmjopen-2014-005245 24993766 10.1136/bmjopen-2014-005245 PMC4091394

[6] Jayasekara H, MacInnis RJ, Hodge AM, Hopper JL, Giles GG, Room R, et al Alcohol consumption for different periods in life, intake pattern over time and all-cause mortality. J Public Health. 2015;37(4): 625–633. 10.1093/pubmed/fdu082 25320075

[7] Gage S. Do the new alcohol guidelines help us understand the risks of drinking? 2016 Jan 08 [cited 16 Feb 2017]. In: The Guardian [Internet]. London. Available from: https://www.theguardian.com/science/sifting-the-evidence/2016/jan/08/do-the-new-alcohol-guidelines-help-us-understand-the-risks-of-drinking

[8] Jivanda T. A bottle of wine a day is not bad for you and abstaining is worse than drinking, scientist claims. 2014 Apr 19 [cited 16 Feb 2017]. In: The Independent [Internet]. London. Available from: http://www.independent.co.uk/life-style/food-and-drink/news/a-bottle-of-wine-a-day-is-not-bad-for-you-and-abstaining-is-worse-than-drinking-scientist-claims-9271010.html

[9] Schütze M, Boeing H, Pischon T, Rehm J, Kehoe T, Gmel G, et al Alcohol attributable burden of incidence of cancer in eight European countries based on results from prospective cohort study. BMJ. 2011;342: d1584 doi: 10.1136/bmj.d1584 21474525 10.1136/bmj.d1584 PMC3072472

[10] WCRF/AICR. Food, Nutrition, Physical Activity, and the Prevention of Cancer: a Global Perspective. Washington, DC: AICR; 2007.

[11] Stockwell T, Zhao J, Panwar S, Roemer A, Naimi T, Chikritzhs T. Do “Moderate” Drinkers Have Reduced Mortality Risk? A Systematic Review and Meta-Analysis of Alcohol Consumption and All-Cause Mortality. J Stud Alcohol Drugs. 2016;77(2): 185–198. doi: 10.15288/jsad.2016.77.185 26997174 10.15288/jsad.2016.77.185 PMC4803651

[12] Friesema IHM, Zwietering PJ, Veenstra MY, Knottnerus JA, Garretsen HFL, Kester ADM, et al The Effect of Alcohol Intake on Cardiovascular Disease and Mortality Disappeared After Taking Lifetime Drinking and Covariates Into Account. Alcohol Clin Exp Res. 2008 4;32(4): 645–651. doi: 10.1111/j.1530-0277.2007.00612.x 18241313 10.1111/j.1530-0277.2007.00612.x

[13] Jayasekara H, English DR, Room R, MacInnis RJ. Alcohol Consumption Over Time and Risk of Death: A Systematic Review and Meta-Analysis. Am J Epidemiol. 2014 5 1;179(9): 1049–1059. doi: 10.1093/aje/kwu028 24670372 10.1093/aje/kwu028

[14] U.S. Department of Health and Human Services and U.S. Department of Agriculture. 2015–2020 Dietary Guidelines for Americans. Washington, DC; 2015.

[15] Prorok PC, Andriole GL, Bresalier RS, Buys SS, Chia D, Crawford DE, et al Design of the prostate, lung, colorectal and ovarian (PLCO) cancer screening trial. Control Clin Trials. 2000 12;21(6 Suppl): 273S–309S. doi: 10.1016/S0197-2456(00)00098-2 11189684 10.1016/s0197-2456(00)00098-2

[16] Subar AF, Thompson FE, Kipnis V, Midthune D, Hurwitz P, McNutt S, et al Comparative Validation of the Block, Willett, and National Cancer Institute Food Frequency Questionnaires: The Eating at America’s Table Study. Am J Epidemiol. 2001 12 15;154(12): 1089–1099. 11744511 10.1093/aje/154.12.1089

[17] Bowman SA, Friday JE, Moshfegh AJ. MyPyramid Equivalents Database, 2.0 for USDA Survey Foods, 2003–2004: Documentation and User Guide. Baltimore; 2004.

[18] World Cancer Research Fund. Global status report on alcohol and health 2014. Geneva; 2014.

[19] Thiébaut ACM, Bénichou J. Choice of time-scale in Cox’s model analysis of epidemiologic cohort data: a simulation study. Stat Med. 2004 12 30;23(24): 3803–3820. doi: 10.1002/sim.2098 15580597 10.1002/sim.2098

[20] Desquilbet L, Mariotti F. Dose-response analyses using restricted cubic spline functions in public health research. Stat Med. 2010;29(9): 1037–1057. doi: 10.1002/sim.3841 20087875 10.1002/sim.3841

[21] Akaike H. Likelihood of a model and information criteria. J Econom. 1981 5;16(1): 3–14.

[22] Cancer Research UK. How alcohol causes cancer [Internet]. [cited 2017 Feb 16]. Available from: http://www.cancerresearchuk.org/about-cancer/causes-of-cancer/alcohol-and-cancer/how-alcohol-causes-cancer

[23] Department of Health. UK Chief Medical Officers’ Alcohol Guidelines Review: Summary of the proposed new guidelines. 2015;(January 2016):7.

[24] Xi B, Veeranki SP, Zhao M, Ma C, Yinkun Y, Jie M. Relationship of Alcohol Consumption to All-Cause, Cardiovascular, and Cancer-Related Mortality in U.S. Adults. J Am Coll Cardiol. 2017 8 22;70(8): 913–922. doi: 10.1016/j.jacc.2017.06.054 28818200 10.1016/j.jacc.2017.06.054

[25] Holmes M V, Dale CE, Zuccolo L, Silverwood RJ, Guo Y, Ye Z, et al Association between alcohol and cardiovascular disease: Mendelian randomisation analysis based on individual participant data. BMJ. 2014 7 10;349: g4164 doi: 10.1136/bmj.g4164 25011450 10.1136/bmj.g4164 PMC4091648

[26] Ruitenberg A, van Swieten JC, Witteman JC, Mehta KM, van Duijn CM, Hofman A, et al Alcohol consumption and risk of dementia: the Rotterdam Study. Lancet. 2002 1 26;359(9303): 281–286. doi: 10.1016/S0140-6736(02)07493-7 11830193 10.1016/S0140-6736(02)07493-7

[27] Naimi TS, Stockwell T, Zhao J, Xuan Z, Dangardt F, Saitz R, et al Selection biases in observational studies affect associations between “moderate” alcohol consumption and mortality. Addiction. 2017 2;112(2): 207–214. doi: 10.1111/add.13451 27316346 10.1111/add.13451

[28] Bergmann MM, Rehm J, Klipstein-Grobusch K, Boeing H, Schütze M, Drogan D, et al The association of pattern of lifetime alcohol use and cause of death in the European Prospective Investigation into Cancer and Nutrition (EPIC) study. Int J Epidemiol. 2013;42(6): 1772–1790. doi: 10.1093/ije/dyt154 24415611 10.1093/ije/dyt154 PMC3887563

[29] Steenland K, Hu S, Walker J. All-cause and cause-specific mortality by socioeconomic status among employed persons in 27 US states, 1984–1997. Am J Public Health. 2004 6;94(6): 1037–1042. 15249312 10.2105/ajph.94.6.1037 PMC1448386

[30] Lee SJ, Sudore RL, Williams BA, Lindquist K, Chen HL, Covinsky KE. Functional Limitations, Socioeconomic Status, and All-Cause Mortality in Moderate Alcohol Drinkers. J Am Geriatr Soc. 2009 6;57(6): 955–962. doi: 10.1111/j.1532-5415.2009.02184.x 19473456 10.1111/j.1532-5415.2009.02184.x PMC2847409

[31] Pinsky P, Miller A, Kramer B, Church T, Reding D, Prorok P, et al Evidence of a Healthy Volunteer Effect in the Prostate, Lung, Colorectal, and Ovarian Cancer Screening Trial. Am J Epidemiol. 2007 2 28;165(8): 874–881. doi: 10.1093/aje/kwk075 17244633 10.1093/aje/kwk075

