# Supplementary material for: The association of lifetime alcohol use with mortality and cancer risk in older adults: A cohort study
Source: PLoS Med. 2018 Jun 19;15(6):e1002585. doi: 10.1371/journal.pmed.1002585 (PMC6007830; doi:10.1371/journal.pmed.1002585)
Supplement: S1 Checklist — (DOCX) [file pmed.1002585.s001.docx]

|  | Item No |  | Section (paragraph number) | |
| --- | --- | --- | --- | --- |
| **Title and abstract** | 1 | (a) | Title page, paragraph 1 | |
|  |  | (*b*) | Abstract | |
| Introduction | | |  |  |
| Background/rationale | 2 |  | Introduction, paragraph 1-4 | |
| Objectives | 3 |  | Introduction, paragraph 5 | |
| Methods | | |  |  |
| Study design | 4 |  | Methods | |
| Setting | 5 |  | Methods, paragraph 1 | |
| Participants | 6 | (*a*) | Methods, paragraph 1 & 9 | |
|  |  | (*b*) | n/a | |
| Variables | 7 |  | Methods, paragraph 2-5 & 13 | |
| Data sources/ measurement | 8* |  | Methods, paragraph 1-7 | |
| Bias | 9 |  | Introduction, paragraph 5. Methods, paragraph 5. | |
| Study size | 10 |  | Methods, paragraph 1 & 9.  Results , paragraph 1 | |
| Quantitative variables | 11 |  | Methods, paragraph 2-5 & 13 | |
| Statistical methods | 12 |  | Methods, paragraph 10-14 | |
| Results | | |  |  |
| Participants | 13* |  | Results, paragraph 1-2 | |
| Descriptive data | 14* | (a) | Results, paragraph 1-2. Table 1 | |
|  |  | (b) | Methods, paragraph 9 | |
|  |  | (c) | Figure 1 | |
| Outcome data | 15* |  | Results, paragraph 3-6 | |
| Main results | 16 |  | Results, paragraph 3-6. Figures 2-5 | |
| Other analyses | 17 |  | Results, paragraph 3-6. Supplementary materials | |
| Discussion | | |  |  |
| Key results | 18 |  | Discussion, paragraph 1 | |
| Limitations | 19 |  | Discussion, paragraph 4-8 | |
| Interpretation | 20 |  | Discussion, paragraph 2-9 | |
| Generalisability | 21 |  | Discussion, paragraph 8 | |
| Other information | | |  |  |
| Funding | 22 |  | Title page, paragraph 7 | |

S1 Checklist. STROBE checklist of items that should be included in reports of cohort studies
