# Supplementary material for: The association of lifetime alcohol use with mortality and cancer risk in older adults: A cohort study
Source: PLoS Med. 2018 Jun 19;15(6):e1002585. doi: 10.1371/journal.pmed.1002585 (PMC6007830; doi:10.1371/journal.pmed.1002585)
Supplement: S1 Text — (DOCX) [file pmed.1002585.s002.docx]

S1 Text. Study protocol when applying for use of the data

The association between alcohol consumption and mortality is complex and controversial. We propose to study the association between lifetime alcohol intake and multiple outcomes, including cancer incidence, all-cause mortality, cancer mortality, cardiovascular mortality and other mortality. Dietary data from both arms of the Prostate, Lung, Colorectal, Ovarian Cancer Screening Trial will be utilised, as the questions in regards to alcohol consumption are comparable in the dietary questionnaire (DQX), given to participants in the intervention arm at baseline, and the dietary history questionnaire (DHQ), given to participants in both arms beginning in 1998 (at approximately 3 years after the baseline for the intervention arm, and at baseline for the control arm participants enrolled after 1998) and . We plan to conduct our primary analyses based on DHQ data but undergo secondary confirming analyses using the DQX data. We will assess whether the associations with mortality or cancer incidence differ between alcohol sources: Beer, Wine and Liquor or between frequency, duration, and amount of consumption. Further sub-group analyses will consider alcohol intake at various ages, drinking status separating former drinkers from long-term non-drinkers, and other demographic and lifestyle factors.

Specific aims

1. Assess the association between alcohol intake and mortality from all-causes and cause-specific mortality.

2. Assess the association between alcohol intake and cancer incidence: all cancer and individual types of cancer.

3. To evaluate whether the associations with mortality or cancer incidence differ between sources of alcohol: Beer, wine and liquor.

4. To assess whether the associations with mortality or cancer incidence differ between frequent light to moderate consumption and infrequent but heavy alcohol consumption.

5. Assess the associations between duration of alcohol use and mortality and cancer incidence.

6. Assess whether the associations between alcohol and mortality or cancer incidence differ after exclusion of former drinkers.

7. Assess whether the associations between alcohol and mortality or cancer incidence differs based on other characteristics such as age, gender, race, BMI, smoking status or educational attainment.
