# Supplementary figures and images for: The association of lifetime alcohol use with mortality and cancer risk in older adults: A cohort study
Source: PLoS Med. 2018 Jun 19;15(6):e1002585. doi: 10.1371/journal.pmed.1002585 (PMC6007830; doi:10.1371/journal.pmed.1002585)

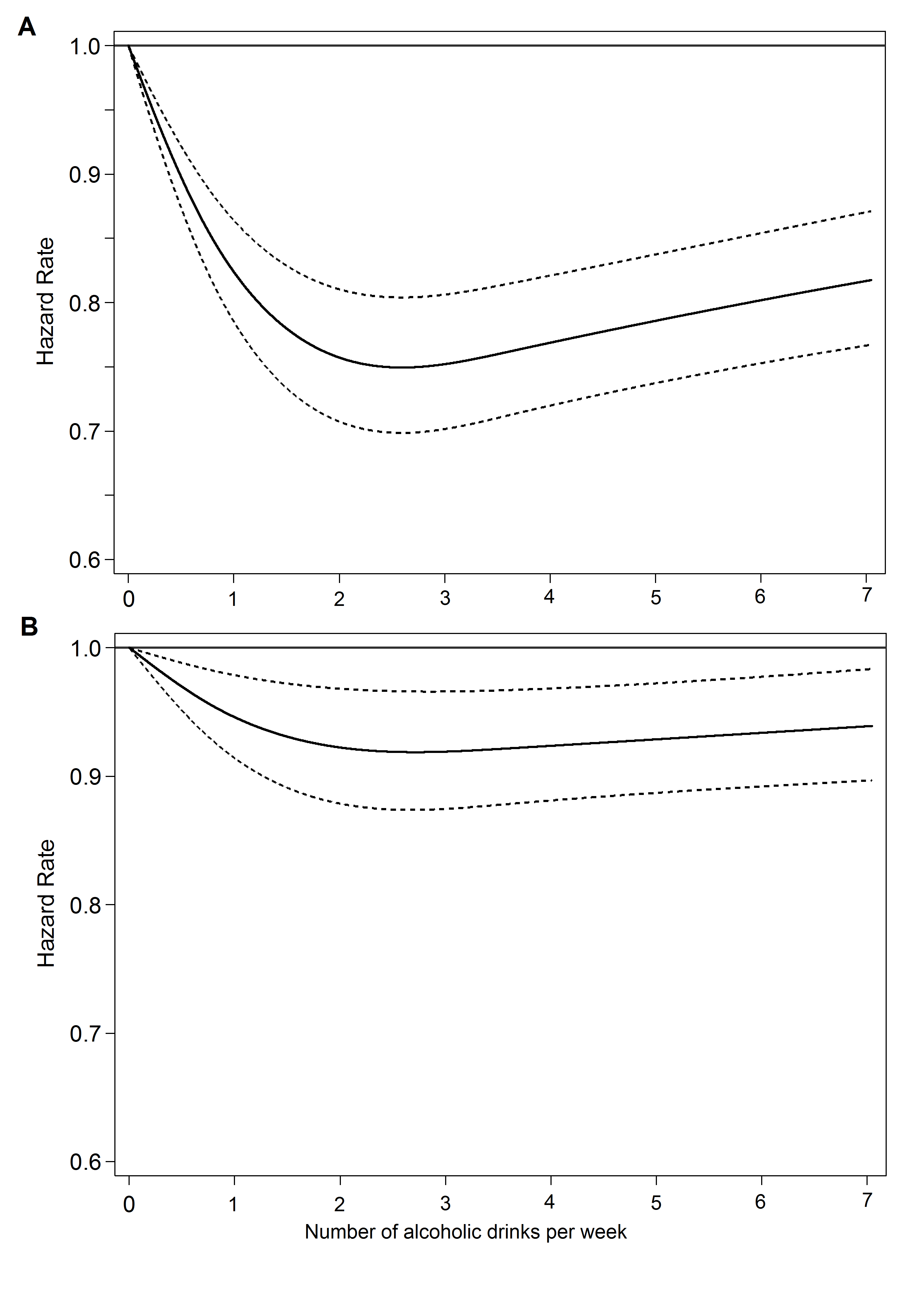

Supplement: S1 Fig — A more detailed figure showing restricted cubic splines for the association between average lifetime alcohol intake up to 7 drinks per week and overall mortality (A, 4 knots) and risk of cancer or death (B, 4 knots/3 splines) in men and women combined (adjusted for gender, study centre, race, BMI, randomisation group, smoking status, year of DHQ completion, marital status, educational attainment, family history of cancer, HRT use [women only], energy intake, red meat intakes per 1,000 kcal, processed meat intakes per 1,000 kcal, coffee intake, fruit and vegetable intake, fibre intake, and calcium intake). BMI, body mass index; DHQ, Diet History Questionnaire; HRT, hormone replacement therapy. (TIF) [file pmed.1002585.s003.tif]

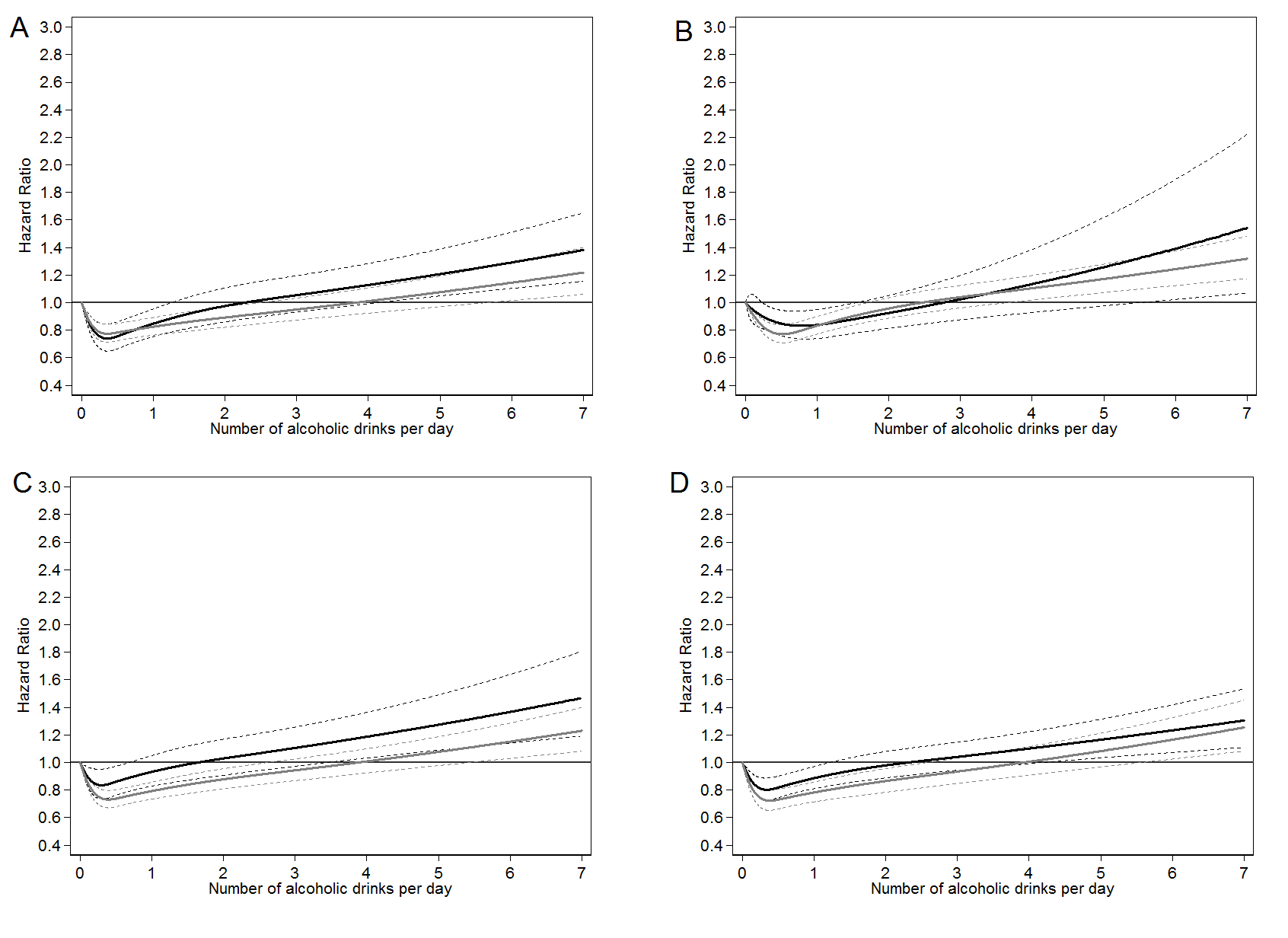

Supplement: S2 Fig — HR (solid lines) and 95% CIs (dashed lines) for the association between average lifetime alcohol intake and overall mortality stratified by (A) age (individuals aged under 65: Black; over 65 years: Grey); (B) Smoking status (Never smokers: Black; Ever smokers: Grey); (C) BMI (Normal BMI: Black; BMI > 25: Grey); (D) Randomisation group (Control group: Black; Intervention group: Grey). BMI, body mass index; CI, confidence interval; HR, hazard ratio. (TIF) [file pmed.1002585.s004.tif]

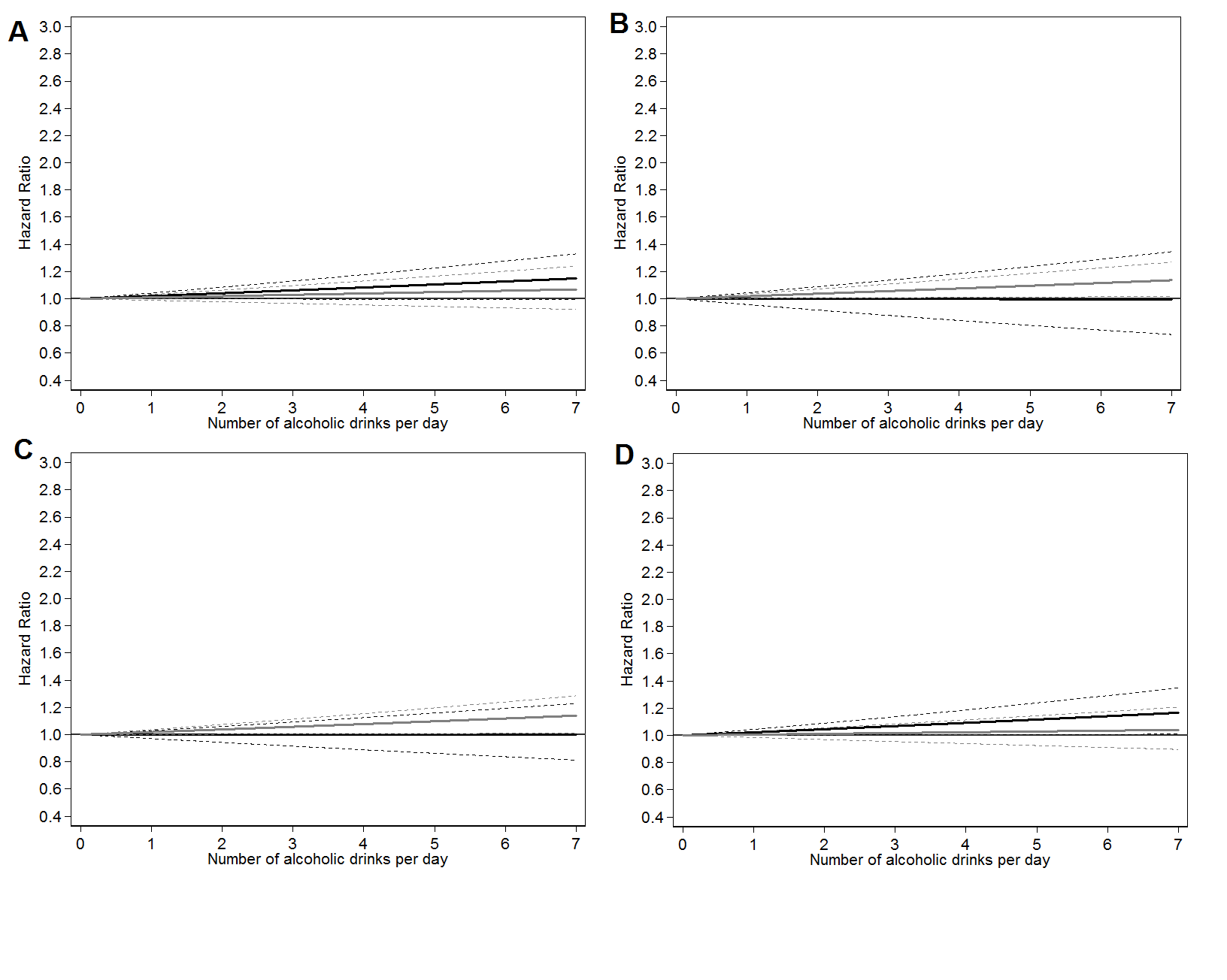

Supplement: S3 Fig — Hazard ratios (solid lines) and 95% confidence intervals (dashed lines) for the association between average lifetime alcohol intake and total cancer risk stratified by (A) age (individuals aged under 65: Black; over 65 years: Grey); (B) Smoking status (Never smokers: Black; Ever smokers: Grey); (C) BMI (Normal BMI: Black; BMI > 25: Grey); (D) Randomisation group (Control group: Black; Intervention group: Grey). BMI, body mass index. (TIF) [file pmed.1002585.s005.tif]

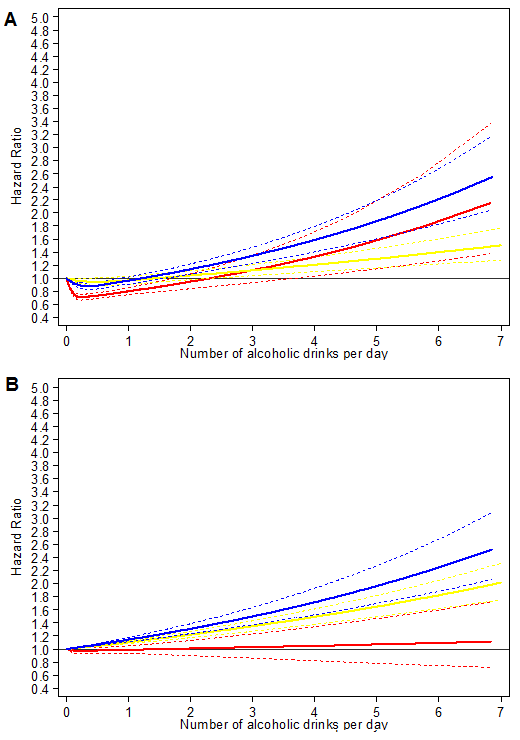

Supplement: S4 Fig — Restricted cubic splines for the association (HR, solid lines) between average lifetime intakes of beer (yellow line), liquor (blue line), and wine (red line) and overall mortality (A, all 1 knots) and total cancer risk (B, wine: 1 knot; Beer and liquor: linear) in men and women combined (adjusted for gender, study centre, race, BMI, randomisation group, smoking status, year of DHQ completion, marital status, educational attainment, family history of cancer, HRT use [women only], energy intake, red meat intakes per 1,000 kcal, processed meat intakes per 1,000 kcal, coffee intake, fruit and vegetable intake, fibre intake, and calcium intake) with corresponding 95% confidence intervals (dashed lines). Akaike's information criterion and likelihood ratio tests were used to investigate if there was a significant improvement to the model fit when fitting restricted cubic spline models, with varying number of knots, compared to a linear model with average lifetime alcohol intake as a continuous variable. BMI, body mass index; DHQ, Diet History Questionnaire; HR, hazard ratio; HRT, hormone replacement therapy. (TIF) [file pmed.1002585.s006.tif]
