# Supplementary material for: The association of lifetime alcohol use with mortality and cancer risk in older adults: A cohort study
Source: PLoS Med. 2018 Jun 19;15(6):e1002585. doi: 10.1371/journal.pmed.1002585 (PMC6007830; doi:10.1371/journal.pmed.1002585)
Supplement: S1 Table — (DOCX) [file pmed.1002585.s007.docx]

S1 Table. The association between average lifetime alcohol and mortality using never drinkers as the reference category in men and women.

|  |  |  | Never drinkers | Infrequent | Light | Somewhat light | Light-moderate | Moderate | Heavy | Very heavy |
| --- | --- | --- | --- | --- | --- | --- | --- | --- | --- | --- |
|  |  |  |  | (0-<1/week) | (1-<3/week) | 3-<5/week | (5-<7/week) | (1-<2/day) | (2-<3/day) | (3+/day) |
| Total mortality | Men | HR (95% CI) | 1.00 (referent) | 0.91 (0.82-1.02) | 0.80 (0.72-0.90) | 0.76 (0.67-0.86) | 0.84 (0.74-0.96) | 0.83 (0.74-0.94) | 0.95 (0.84-1.09) | 1.09 (0.96-1.24) |
|  | Women | HR (95% CI) | 1.00 (referent) | 0.95 (0.86-1.05) | 0.78 (0.69-0.87) | 0.78 (0.67-0.91) | 0.85 (0.71-1.02) | 0.86 (0.72-1.02) | 1.07 (0.82-1.39) | 1.54 (1.16-2.06) |
|  |  |  |  |  |  |  |  |  |  |  |
| Cardiovascular-related mortality | Men | HR (95% CI) | 1.00 (referent) | 0.83 (0.68-1.00) | 0.85 (0.70-1.03) | 0.76 (0.61-0.93) | 0.82 (0.66-1.02) | 0.83 (0.68-1.02) | 0.96 (0.77-1.20) | 1.05 (0.85-1.31) |
|  | Women | HR (95% CI) | 1.00 (referent) | 0.88 (0.73-1.05) | 0.63 (0.51-0.80) | 0.58 (0.43-0.78) | 0.80 (0.57-1.12) | 0.60 (0.42-0.86) | 0.80 (0.46-1.37) | 1.08 (0.58-2.02) |
|  |  |  |  |  |  |  |  |  |  |  |
| Cancer-related mortality | Men | HR (95% CI) | 1.00 (referent) | 0.94 (0.76-1.16) | 0.81 (0.66-1.00) | 0.82 (0.66-1.02) | 0.92 (0.73-1.16) | 0.85 (0.68-1.05) | 1.00 (0.79-1.27) | 1.00 (0.79-1.26) |
|  | Women | HR (95% CI) | 1.00 (referent) | 1.01 (0.84-1.21) | 0.97 (0.79-1.18) | 0.88 (0.69-1.13) | 0.93 (0.69-1.26) | 1.13 (0.86-1.48) | 1.47 (1.00-2.16) | 1.18 (0.69-2.02) |
|  |  |  |  |  |  |  |  |  |  |  |
| Mortality from accidents, suicide & homicide | Men | HR (95% CI) | 1.00 (referent) | 1.14 (0.69-1.87) | 0.83 (0.49-1.39) | 0.74 (0.42-1.30) | 1.05 (0.59-1.85) | 0.86 (0.50-1.48) | 0.96 (0.52-1.78) | 1.21 (0.67-2.18) |
|  | Women | HR (95% CI) | 1.00 (referent) | 0.86 (0.51-1.45) | 1.00 (0.57-1.79) | 1.47 (0.77-2.79) | 1.13 (0.48-2.65) | 0.65 (0.24-1.77) | 0.00 (0.00-0.00) | 2.00 (0.46-8.78) |
|  |  |  |  |  |  |  |  |  |  |  |
| Mortality from other causes | Men | HR (95% CI) | 1.00 (referent) | 0.96 (0.78-1.18) | 0.74 (0.60-0.92) | 0.72 (0.57-0.91) | 0.75 (0.58-0.96) | 0.81 (0.65-1.02) | 0.89 (0.69-1.15) | 1.25 (0.98-1.58) |
|  | Women | HR (95% CI) | 1.00 (referent) | 0.97 (0.81-1.16) | 0.64 (0.51-0.80) | 0.76 (0.58-0.99) | 0.77 (0.55-1.07) | 0.84 (0.61-1.14) | 1.02 (0.63-1.65) | 2.74 (1.79-4.19) |
|  |  |  |  |  |  |  |  |  |  |  |
| Mortality from alcohol-related cancers | Men | HR (95% CI) | 1.00 (referent) | 0.94 (0.53-1.67) | 0.85 (0.48-1.51) | 0.84 (0.46-1.53) | 0.95 (0.51-1.77) | 0.90 (0.50-1.61) | 1.40 (0.76-2.56) | 1.69 (0.94-3.04) |
|  | Women | HR (95% CI) | 1.00 (referent) | 1.20 (0.79-1.83) | 1.25 (0.78-2.01) | 1.31 (0.74-2.34) | 0.87 (0.38-2.03) | 1.42 (0.70-2.85) | 1.91 (0.66-5.54) | 2.54 (0.76-8.50) |
|  |  |  |  |  |  |  |  |  |  |  |
| Mortality from other cancers | Men | HR (95% CI) | 1.00 (referent) | 0.94 (0.75-1.18) | 0.80 (0.64-1.01) | 0.82 (0.64-1.04) | 0.92 (0.71-1.17) | 0.84 (0.66-1.06) | 0.94 (0.73-1.22) | 0.89 (0.68-1.15) |
|  | Women | HR (95% CI) | 1.00 (referent) | 0.97 (0.79-1.18) | 0.91 (0.73-1.14) | 0.82 (0.62-1.07) | 0.93 (0.68-1.28) | 1.09 (0.81-1.45) | 1.43 (0.95-2.17) | 1.08 (0.59-1.96) |
|  |  |  |  |  |  |  |  |  |  |  |
| Total cancer risk | Men | HR (95% CI) | 1.00 (referent) | 1.05 (0.95-1.16) | 1.03 (0.93-1.15) | 1.04 (0.93-1.16) | 1.09 (0.97-1.22) | 1.03 (0.92-1.15) | 1.10 (0.98-1.25) | 1.11 (0.98-1.25) |
|  | Women | HR (95% CI) | 1.00 (referent) | 1.08 (0.98-1.18) | 1.05 (0.95-1.17) | 1.09 (0.96-1.23) | 1.11 (0.95-1.29) | 1.16 (1.00-1.35) | 1.14 (0.89-1.45) | 1.08 (0.78-1.49) |
|  |  |  |  |  |  |  |  |  |  |  |
| Alcohol-related cancer risk | Men | HR (95% CI) | 1.00 (referent) | 1.06 (0.76-1.48) | 0.90 (0.64-1.26) | 0.96 (0.68-1.37) | 1.35 (0.95-1.92) | 1.01 (0.71-1.43) | 1.31 (0.90-1.89) | 1.66 (1.16-2.38) |
|  | Women | HR (95% CI) | 1.00 (referent) | 1.10 (0.96-1.26) | 1.06 (0.91-1.24) | 1.16 (0.97-1.39) | 1.26 (1.02-1.57) | 1.18 (0.94-1.47) | 1.29 (0.90-1.85) | 1.32 (0.82-2.12) |
|  |  |  |  |  |  |  |  |  |  |  |
| Other cancer risk | Men | HR (95% CI) | 1.00 (referent) | 1.00 (0.92-1.09) | 0.96 (0.88-1.04) | 0.93 (0.85-1.02) | 0.98 (0.90-1.08) | 0.96 (0.88-1.05) | 1.04 (0.95-1.15) | 1.13 (1.03-1.25) |
|  | Women | HR (95% CI) | 1.00 (referent) | 1.05 (0.93-0.00) | 1.03 (0.89-1.19) | 1.02 (0.86-1.21) | 0.98 (0.79-1.21) | 1.14 (0.94-1.39) | 1.02 (0.73-1.42) | 0.91 (0.58-1.43) |
|  |  |  |  |  |  |  |  |  |  |  |
| Risk of cancer or death | Men | HR (95% CI) | 1.00 (referent) | 1.00 (0.92-1.09) | 0.96 (0.88-1.04) | 0.93 (0.85-1.02) | 0.98 (0.90-1.08) | 0.96 (0.88-1.05) | 1.04 (0.95-1.15) | 1.13 (1.03-1.25) |
|  | Women | HR (95% CI) | 1.00 (referent) | 1.02 (0.95-1.10) | 0.92 (0.84-1.00) | 0.95 (0.86-1.06) | 1.00 (0.88-1.13) | 1.00 (0.89-1.14) | 1.03 (0.84-1.27) | 1.34 (1.06-1.69) |

All models adjusted for: Study centre, race (Non-hispanic white, Non-hispanic black, Asian, Other) BMI, randomisation group (Control, intervention), smoking status by pack-years (Never, former low-pack-years, former high pack-years, current low pack-years, current high pack-years), year of DHQ completion, marital status (Married, widowed, divorced, separated, never married) educational attainment (<11 years, 12years/completed high school, some college/post high school, graduate/postgraduate), family history of cancer (Yes, no), HRT use (women only, current, former never), coffee intake (cups/day), energy intake, red meat intakes/1000kcal, processed meat intakes/1000kcal, fruit and vegetable intake/1000kcal (MPED), dietary fibre intake per 1000kcal and total calcium intake/1000kcal (diet & supplements).
