# Supplementary material for: The association of lifetime alcohol use with mortality and cancer risk in older adults: A cohort study
Source: PLoS Med. 2018 Jun 19;15(6):e1002585. doi: 10.1371/journal.pmed.1002585 (PMC6007830; doi:10.1371/journal.pmed.1002585)
Supplement: S2 Table — (DOCX) [file pmed.1002585.s008.docx]

S2 Table. The association between average lifetime alcohol and cancer outcomes using light drinkers as the reference category in men and women.

|  |  |  | Never drinkers | | Infrequent | | Light | | Somewhat light | | Light-moderate | | Moderate | | Heavy | | Very heavy | |  |
| --- | --- | --- | --- | --- | --- | --- | --- | --- | --- | --- | --- | --- | --- | --- | --- | --- | --- | --- | --- |
|  |  |  |  | | (0-<1/week) | | (1-<3/week) | | 3-<5/week | | (5-<7/week) | | (1-<2/day) | | (2-<3/day) | | (3+/day) | |  |
| Total mortality | Men | HR (95% CI) | | 1.25 (1.11-1.40) | | 1.14 (1.04-1.24) | | 1.00 (referent) | | 0.95 (0.87-1.05) | | 1.05 (0.95-1.16) | | 1.03 (0.95-1.13) | | 1.19 (1.07-1.32) | | 1.36 (1.23-1.50) | |
|  | Women | HR (95% CI) | | 1.29 (1.14-1.46) | | 1.23 (1.12-1.35) | | 1.00 (referent) | | 1.01 (0.88-1.15) | | 1.10 (0.93-1.30) | | 1.11 (0.95-1.30) | | 1.38 (1.07-1.78) | | 1.99 (1.51-2.64) | |
| Cardiovascular-related mortality | Men | HR (95% CI) | | 1.18 (0.97-1.43) | | 0.97 (0.84-1.13) | | 1.00 (referent) | | 0.89 (0.76-1.04) | | 0.97 (0.81-1.15) | | 0.98 (0.85-1.14) | | 1.13 (0.95-1.34) | | 1.24 (1.05-1.47) | |
|  | Women | HR (95% CI) | | 1.58 (1.26-1.98) | | 1.38 (1.15-1.65) | | 1.00 (referent) | | 0.91 (0.68-1.20) | | 1.26 (0.91-1.73) | | 0.95 (0.67-1.33) | | 1.26 (0.74-2.14) | | 1.71 (0.92-3.16) | |
| Cancer-related mortality | Men | HR (95% CI) | | 1.23 (1.00-1.53) | | 1.16 (0.99-1.36) | | 1.00 (referent) | | 1.01 (0.86-1.19) | | 1.13 (0.95-1.35) | | 1.04 (0.89-1.22) | | 1.24 (1.04-1.48) | | 1.23 (1.03-1.47) | |
|  | Women | HR (95% CI) | | 1.03 (0.84-1.26) | | 1.04 (0.90-1.20) | | 1.00 (referent) | | 0.91 (0.74-1.13) | | 0.97 (0.74-1.26) | | 1.16 (0.92-1.47) | | 1.52 (1.06-2.18) | | 1.21 (0.72-2.05) | |
| Mortality from accidents, suicide & homicide | Men | HR (95% CI) | | 1.41 (1.27-0.81) | | 1.99 (1.04-0.68) | | 1.00 (referent) | | 1.93 (1.46-0.91) | | 2.34 (0.81-1.99) | | 1.04 (0.68-1.58) | | 1.17 (0.71-1.93) | | 1.46 (0.91-2.34) | |
|  | Women | HR (95% CI) | | 2.48 (1.08-0.50) | | 2.34 (0.60-0.23) | | 1.00 (referent) | | 0.00 (1.79-0.42) | | 7.59 (0.50-2.34) | | 0.60 (0.23-1.54) | | 0.00 (0.00-0.00) | | 1.79 (0.42-7.59) | |
| Mortality from other causes | Men | HR (95% CI) | | 1.17 (1.01-0.83) | | 1.23 (1.09-0.92) | | 1.00 (referent) | | 1.47 (1.68-1.40) | | 2.03 (0.83-1.23) | | 1.09 (0.92-1.30) | | 1.20 (0.98-1.47) | | 1.68 (1.40-2.03) | |
|  | Women | HR (95% CI) | | 1.51 (1.18-0.86) | | 1.63 (1.26-0.94) | | 1.00 (referent) | | 2.40 (3.59-2.36) | | 5.45 (0.86-1.63) | | 1.26 (0.94-1.69) | | 1.49 (0.93-2.40) | | 3.59 (2.36-5.45) | |
| Mortality from alcohol-related cancers | Men | HR (95% CI) | | 1.52 (1.12-0.71) | | 1.76 (1.06-0.71) | | 1.00 (referent) | | 2.52 (1.98-1.32) | | 2.98 (0.71-1.76) | | 1.06 (0.71-1.58) | | 1.64 (1.07-2.52) | | 1.98 (1.32-2.98) | |
|  | Women | HR (95% CI) | | 1.74 (0.69-0.31) | | 1.53 (1.14-0.60) | | 1.00 (referent) | | 4.25 (2.10-0.65) | | 6.86 (0.31-1.53) | | 1.14 (0.60-2.15) | | 1.52 (0.55-4.25) | | 2.10 (0.65-6.86) | |
| Mortality from other cancers | Men | HR (95% CI) | | 1.21 (1.14-0.94) | | 1.37 (1.04-0.88) | | 1.00 (referent) | | 1.42 (1.10-0.90) | | 1.34 (0.94-1.37) | | 1.04 (0.88-1.23) | | 1.17 (0.96-1.42) | | 1.10 (0.90-1.34) | |
|  | Women | HR (95% CI) | | 1.12 (1.01-0.76) | | 1.34 (1.17-0.91) | | 1.00 (referent) | | 2.23 (1.09-0.61) | | 1.96 (0.76-1.34) | | 1.17 (0.91-1.51) | | 1.51 (1.03-2.23) | | 1.09 (0.61-1.96) | |
| Total cancer risk | Men | HR (95% CI) | | 1.09 (1.05-0.97) | | 1.15 (1.00-0.92) | | 1.00 (referent) | | 1.17 (1.07-0.98) | | 1.18 (0.97-1.15) | | 1.00 (0.92-1.08) | | 1.07 (0.97-1.17) | | 1.07 (0.98-1.18) | |
|  | Women | HR (95% CI) | | 1.15 (1.06-0.92) | | 1.21 (1.11-0.97) | | 1.00 (referent) | | 1.37 (1.03-0.75) | | 1.41 (0.92-1.21) | | 1.11 (0.97-1.26) | | 1.09 (0.86-1.37) | | 1.03 (0.75-1.41) | |
| Alcohol-related cancer risk | Men | HR (95% CI) | | 1.39 (1.50-1.16) | | 1.95 (1.13-0.88) | | 1.00 (referent) | | 1.93 (1.85-1.42) | | 2.41 (1.16-1.95) | | 1.13 (0.88-1.44) | | 1.46 (1.10-1.93) | | 1.85 (1.42-2.41) | |
|  | Women | HR (95% CI) | | 1.27 (1.19-0.98) | | 1.45 (1.11-0.91) | | 1.00 (referent) | | 1.71 (1.24-0.78) | | 1.97 (0.98-1.45) | | 1.11 (0.91-1.35) | | 1.21 (0.86-1.71) | | 1.24 (0.78-1.97) | |
| Other cancer risk | Men | HR (95% CI) | | 1.08 (1.01-0.92) | | 1.10 (0.98-0.91) | | 1.00 (referent) | | 1.14 (0.99-0.90) | | 1.10 (0.92-1.10) | | 0.98 (0.91-1.07) | | 1.03 (0.93-1.14) | | 0.99 (0.90-1.10) | |
|  | Women | HR (95% CI) | | 1.15 (0.95-0.79) | | 1.15 (1.11-0.93) | | 1.00 (referent) | | 1.37 (0.89-0.58) | | 1.38 (0.79-1.15) | | 1.11 (0.93-1.32) | | 1.00 (0.73-1.37) | | 0.89 (0.58-1.38) | |
| Risk of cancer or death | Men | HR (95% CI) | | 1.04 (1.03-0.96) | | 1.11 (1.00-0.94) | | 1.00 (referent) | | 1.18 (1.19-1.10) | | 1.28 (0.96-1.11) | | 1.00 (0.94-1.07) | | 1.09 (1.01-1.18) | | 1.19 (1.10-1.28) | |
|  | Women | HR (95% CI) | | 1.14 (1.09-0.97) | | 1.22 (1.10-0.98) | | 1.00 (referent) | | 1.37 (1.47-1.17) | | 1.84 (0.97-1.22) | | 1.10 (0.98-1.23) | | 1.13 (0.93-1.37) | | 1.47 (1.17-1.84) | |

All models adjusted for: Study centre, race (Non-hispanic white, Non-hispanic black, Asian, Other) BMI, randomisation group (Control, intervention), smoking status by pack-years (Never, former low-pack-years, former high pack-years, current low pack-years, current high pack-years), year of DHQ completion, marital status (Married, widowed, divorced, separated, never married) educational attainment (<11 years, 12years/completed high school, some college/post high school, graduate/postgraduate), family history of cancer (Yes, no), HRT use (women only, current, former never), coffee intake (cups/day), energy intake, red meat intakes/1000kcal, processed meat intakes/1000kcal, fruit and vegetable intake/1000kcal (MPED), dietary fibre intake per 1000kcal and total calcium intake/1000kcal (diet & supplements).
