# Supplementary material for: The association of lifetime alcohol use with mortality and cancer risk in older adults: A cohort study
Source: PLoS Med. 2018 Jun 19;15(6):e1002585. doi: 10.1371/journal.pmed.1002585 (PMC6007830; doi:10.1371/journal.pmed.1002585)
Supplement: S3 Table — (DOCX) [file pmed.1002585.s009.docx]

S3 Table. The association between average lifetime alcohol intakes and risk of incident cancers (by type) using never drinkers as the reference category after adjustment for selected confounders.

|  |  | Never drinkers | Infrequent | Light | Somewhat light | Light-moderate | Moderate | Heavy | Very heavy |
| --- | --- | --- | --- | --- | --- | --- | --- | --- | --- |
|  |  |  | (0-<1/week) | (1-<3/week) | 3-<5/week | (5-<7/week) | (1-<2/day) | (2-<3/day) | (3+/day) |
| **Combined (men and women)** | |  |  |  |  |  |  |  |  |
|  | Person-years | 97416.2 | 218856.8 | 167636.1 | 93358.1 | 57539.2 | 81955.4 | 35295.3 | 32995.2 |
| Colorectal cancer | Cancers | 103 | 269 | 183 | 107 | 90 | 91 | 53 | 48 |
|  | HR (95% CI) | 1.00 (referent) | 1.12 (0.88-1.42) | 0.98 (0.76-1.28) | 1.01 (0.75-1.35) | 1.35 (0.99-1.84) | 0.94 (0.69-1.29) | 1.25 (0.87-1.80) | 1.21 (0.83-1.76) |
|  |  |  |  |  |  |  |  |  |  |
| Esophageal cancer | Cancers | 6 | 23 | 17 | 12 | 13 | 21 | 13 | 18 |
|  | HR (95% CI) | 1.00 (referent) | 1.24 (0.49-3.12) | 1.05 (0.40-2.79) | 1.21 (0.43-3.38) | 1.99 (0.72-5.55) | 2.07 (0.78-5.49) | 2.85 (1.01-8.01) | 3.99 (1.47-10.82) |
|  |  |  |  |  |  |  |  |  |  |
| Liver cancer | Cancers | 8 | 9 | 12 | 7 | 6 | 10 | 7 | 14 |
|  | HR (95% CI) | 1.00 (referent) | 0.43 (0.16-1.17) | 0.74 (0.28-1.97) | 0.71 (0.24-2.14) | 0.96 (0.30-3.04) | 1.06 (0.37-3.05) | 1.67 (0.53-5.22) | 3.53 (1.26-9.87) |
|  |  |  |  |  |  |  |  |  |  |
| Head & neck cancer | Cancers | 13 | 25 | 28 | 21 | 30 | 36 | 26 | 41 |
|  | HR (95% CI) | 1.00 (referent) | 0.61 (0.31-1.22) | 0.75 (0.37-1.51) | 0.88 (0.42-1.85) | 1.88 (0.92-3.81) | 1.43 (0.71-2.88) | 2.12 (1.01-4.43) | 3.12 (1.54-6.34) |
|  |  |  |  |  |  |  |  |  |  |
| Lung cancer | Cancers | 73 | 274 | 246 | 188 | 137 | 221 | 130 | 120 |
|  | HR (95% CI) | 1.00 (referent) | 0.87 (0.66-1.13) | 0.80 (0.61-1.05) | 0.95 (0.72-1.27) | 1.03 (0.76-1.39) | 1.03 (0.78-1.37) | 1.21 (0.89-1.64) | 1.04 (0.76-1.42) |
|  |  |  |  |  |  |  |  |  |  |
| **Male Cancers** | |  |  |  |  |  |  |  |  |
|  | Person-years | 29098.5 | 57782.7 | 69886.2 | 52464.5 | 37986.3 | 61267.4 | 29998.5 | 30084.2 |
| Prostate cancer | Cancers | 331 | 602 | 762 | 549 | 399 | 640 | 285 | 283 |
|  | HR (95% CI) | 1.00 (referent) | 1.02 (0.88-1.18) | 1.09 (0.94-1.26) | 1.06 (0.91-1.24) | 1.09 (0.92-1.28) | 1.09 (0.94-1.27) | 1.04 (0.87-1.24) | 1.07 (0.90-1.28) |
|  |  |  |  |  |  |  |  |  |  |
| **Female cancers** | |  |  |  |  |  |  |  |  |
|  | Person-years | 68317.7 | 161074.0 | 97749.9 | 40893.5 | 19552.9 | 20688.0 | 5296.9 | 2911.0 |
| Breast cancer | Cancers | 319 | 831 | 517 | 253 | 117 | 126 | 29 | 19 |
|  | HR (95% CI) | 1.00 (referent) | 1.11 (0.96-1.27) | 1.13 (0.96-1.31) | 1.31 (1.09-1.57) | 1.25 (1.00-1.57) | 1.26 (1.01-1.58) | 1.15 (0.78-1.70) | 1.43 (0.89-2.29) |
|  |  |  |  |  |  |  |  |  |  |
| Ovarian cancer | Cancers | 30 | 78 | 66 | 17 | 11 | 14 | 3 | 2 |
|  | HR (95% CI) | 1.00 (referent) | 1.18 (0.75-1.86) | 1.59 (0.98-2.57) | 0.94 (0.49-1.78) | 1.22 (0.58-2.56) | 1.51 (0.75-3.02) | 1.27 (0.37-4.30) | 1.62 (0.37-7.03) |

All models adjusted for: Study centre, race (Non-hispanic white, Non-hispanic black, Asian, Other) BMI, randomisation group (Control, intervention), smoking status by pack-years (Never, former low-pack-years, former high pack-years, current low pack-years, current high pack-years), year of DHQ completion, marital status (Married, widowed, divorced, separated, never married) educational attainment (<11 years, 12years/completed high school, some college/post high school, graduate/postgraduate), family history of cancer (Yes, no), HRT use (women only, current, former never), coffee intake (cups/day), energy intake, red meat intakes/1000kcal, processed meat intakes/1000kcal, fruit and vegetable intake/1000kcal (MPED), dietary fibre intake per 1000kcal and total calcium intake/1000kcal (diet & supplements).
